# Pyruvate metabolism dictates fibroblast sensitivity to GLS1 inhibition during fibrogenesis

**DOI:** 10.1172/jci.insight.178453

**Published:** 2024-08-13

**Authors:** Greg Contento, Jo-Anne A.M. Wilson, Brintha Selvarajah, Manuela Platé, Delphine Guillotin, Valle Morales, Marcello Trevisani, Vanessa Pitozzi, Katiuscia Bianchi, Rachel C. Chambers

**Affiliations:** 1 Centre for Inflammation and Tissue Repair, UCL Respiratory, University College London, London, United Kingdom.; 2 Centre for Molecular Oncology, Barts Cancer Institute, Queen Mary University of London, John Vane Science Centre, London, United Kingdom.; 3 Corporate Pre-Clinical R&D, Chiesi Farmaceutici S.p.A., Parma, Italy.

**Keywords:** Cell biology, Metabolism, Collagens, Fibrosis, Glucose metabolism

## Abstract

Fibrosis is a chronic disease characterized by excessive extracellular matrix production, which leads to disruption of organ function. Fibroblasts are key effector cells of this process, responding chiefly to the pleiotropic cytokine transforming growth factor–β_1_ (TGF-β_1_), which promotes fibroblast to myofibroblast differentiation. We found that extracellular nutrient availability profoundly influenced the TGF-β_1_ transcriptome of primary human lung fibroblasts and that biosynthesis of amino acids emerged as a top enriched TGF-β_1_ transcriptional module. We subsequently uncovered a key role for pyruvate in influencing glutaminase (GLS1) inhibition during TGF-β_1_–induced fibrogenesis. In pyruvate-replete conditions, GLS1 inhibition was ineffective in blocking TGF-β_1_–induced fibrogenesis, as pyruvate can be used as the substrate for glutamate and alanine production via glutamate dehydrogenase (GDH) and glutamic-pyruvic transaminase 2 (GPT2), respectively. We further show that dual targeting of either GPT2 or GDH in combination with GLS1 inhibition was required to fully block TGF-β_1_–induced collagen synthesis. These findings embolden a therapeutic strategy aimed at additional targeting of mitochondrial pyruvate metabolism in the presence of a glutaminolysis inhibitor to interfere with the pathological deposition of collagen in the setting of pulmonary fibrosis and potentially other fibrotic conditions.

## Abstract 2

● GLS1 inhibition is ineffective in pyruvate replete conditions and additional targetting of GDH or GPT2 is required to effectively prevent pathological levels of collagen synthesis.

## Introduction

Fibrosis is the concluding pathological outcome and major cause of morbidity and mortality in a number of common chronic inflammatory, immune-mediated, and metabolic diseases (1). The progressive and relentless deposition of a collagen-rich extracellular matrix (ECM) is the cornerstone of the fibrotic response and often culminates in organ failure and premature death. Despite intense research efforts, there remains a paucity of effective treatment options. Idiopathic pulmonary fibrosis (IPF) represents the most rapidly progressive and lethal of all fibrotic diseases and is associated with a dismal median survival of 3.5 years from diagnosis (2, 3). Although the approval of pirfenidone and nintedanib for the treatment of IPF signaled a watershed moment for the development of antifibrotic therapeutics, these agents slow but do not halt disease progression (4, 5). There remains a pressing need to identify novel antifibrotic therapeutic strategies.

Activated fibroblasts and myofibroblasts are the key effector cells of the fibrogenic response and are responsible for the synthesis and deposition of collagen and other ECM proteins during normal tissue repair, as well as in pathological tissue fibrosis (6). These cells can originate from several sources, including recruited and resident fibroblasts, which differentiate in response to pro-fibrotic stimuli released at sites of acute and chronic injury, primarily the potent pro-fibrotic cytokine, transforming growth factor–β_1_ (TGF-β_1_) (7). Myofibroblasts are also expanded as a result of the stromal reaction in cancer, and current evidence provides strong support that the density of stromal myofibroblasts is associated with poor survival in solid cancers (8).

Metabolic reprogramming is a hallmark of cancer and supports the requirements of exponential growth and proliferation, as well as metastasis as the cancer progresses (9). This has led to the development of several strategies aimed at exploiting the metabolic vulnerabilities of cancer cells as a potential therapeutic strategy, with several metabolism-targeting therapeutic agents demonstrating good tolerability and efficacy in recent clinical trials. One of these is CB-839, a nanomolar-range glutaminase 1 (GLS1) inhibitor that is being investigated in several trials (NCT02071862, NCT02071927, NCT02071888, among others). GLS1 is the rate-limiting enzyme for glutaminolysis, a process that converts glutamine into the anaplerotic intermediate α-ketoglutarate. This process is important for the generation of glutamate, which is a major amino group donor for aminotransferase reactions, producing amino acids such as alanine or aspartate.

Metabolic reprogramming is also increasingly recognized as an important feature of pathological fibrosis (10, 11). Indeed, there is accumulating in vitro evidence that fibroblast differentiation in response to TGF-β_1_ is accompanied by the reconfiguration of fibroblast metabolic networks to meet the biosynthetic and energetic needs of cell differentiation and ECM production. Identifying and targeting the synthetic vulnerabilities of myofibroblasts may therefore similarly herald a new era for the development of future antifibrotic therapeutic approaches.

Glucose and glutamine metabolism are highly modulated following TGF-β_1_ stimulation, yet the concentrations of these 2 major carbon and nitrogen sources are highly variable among tissue culture media, which may not only influence intracellular metabolic networks but also perturb the balance between gene expression and metabolism (12, 13). Several studies in multiple disease models have shown that altering the concentrations of these key nutrients can be therapeutically beneficial (14, 15). This strategy is particularly promising in disease contexts where the immediate nutrient supply is unknown or variable throughout pathogenesis, such as a poorly vascularized but growing tumor or fibroblastic foci, the hallmark lesion associated with IPF.

To identify antifibrotic strategies based on the exploitation of key metabolic vulnerabilities of myofibroblasts, we investigated the influence of nutrient availability on the TGF-β_1_–induced transcriptome and collagen synthetic capability of primary human lung fibroblasts. We found that the TGF-β_1_–regulated transcriptome is highly sensitive to nutrient availability and further discovered a role for pyruvate in terms of dictating the sensitivity of fibroblasts to GLS1 inhibition during fibrogenesis. This effect was traced to a superior ability for exogenous pyruvate over endogenously produced pyruvate from glucose to maintain amino acid biosynthesis through the enzymes glutamic-pyruvic transaminase 2 (GPT2) and glutamate dehydrogenase (GDH). The findings reported here build a case for the potential of antifibrotic approaches involving the inhibition of GLS1 in combination with inhibition of either GPT2 or GDH to target key metabolic vulnerabilities of hypersynthetic activated fibroblasts during fibrogenesis.

## Results

### The TGF-β_1_–induced transcriptome is modulated by glucose and glutamine metabolism.

TGF-β_1_ is known to promote a wide-ranging transcriptional response in multiple cell types and has been shown to increase chromatin accessibility of genomic enhancer regions by as much as 80% (19). Chromatin accessibility is intimately dependent on metabolic reactions, including epigenetic acetylation, methylation, and demethylation, which are all highly dependent on glucose and glutamine metabolism. However, the concentrations of glucose and glutamine vary widely in the culture media employed across mechanistic studies aimed toward understanding these processes in health and disease. DMEM is a common media formulation for the cultivation of fibroblasts and traditionally contains 25 mM glucose and 2 mM glutamine (termed DMEM^hi^), far higher than the approximate 5 mM glucose and 0.7 mM glutamine (termed DMEM^lo^) present in DMEM formulations aimed at replicating human sera levels. Pyruvate concentrations also differ from absent (DMEM^lo^) to 1 mM (DMEM^hi^). To investigate the effects of media composition on the TGF-β_1_–induced transcriptome, we compared an RNA-Seq dataset of primary human lung fibroblasts (pHLFs) cultured in DMEM^lo^ with a previously published dataset of the same cells exposed to the same concentration of TGF-β_1_ (1 ng/mL) in DMEM^hi^ from our laboratory (NCBI Gene Expression Omnibus [GEO] accession GSE102674).

**Figure 1:**
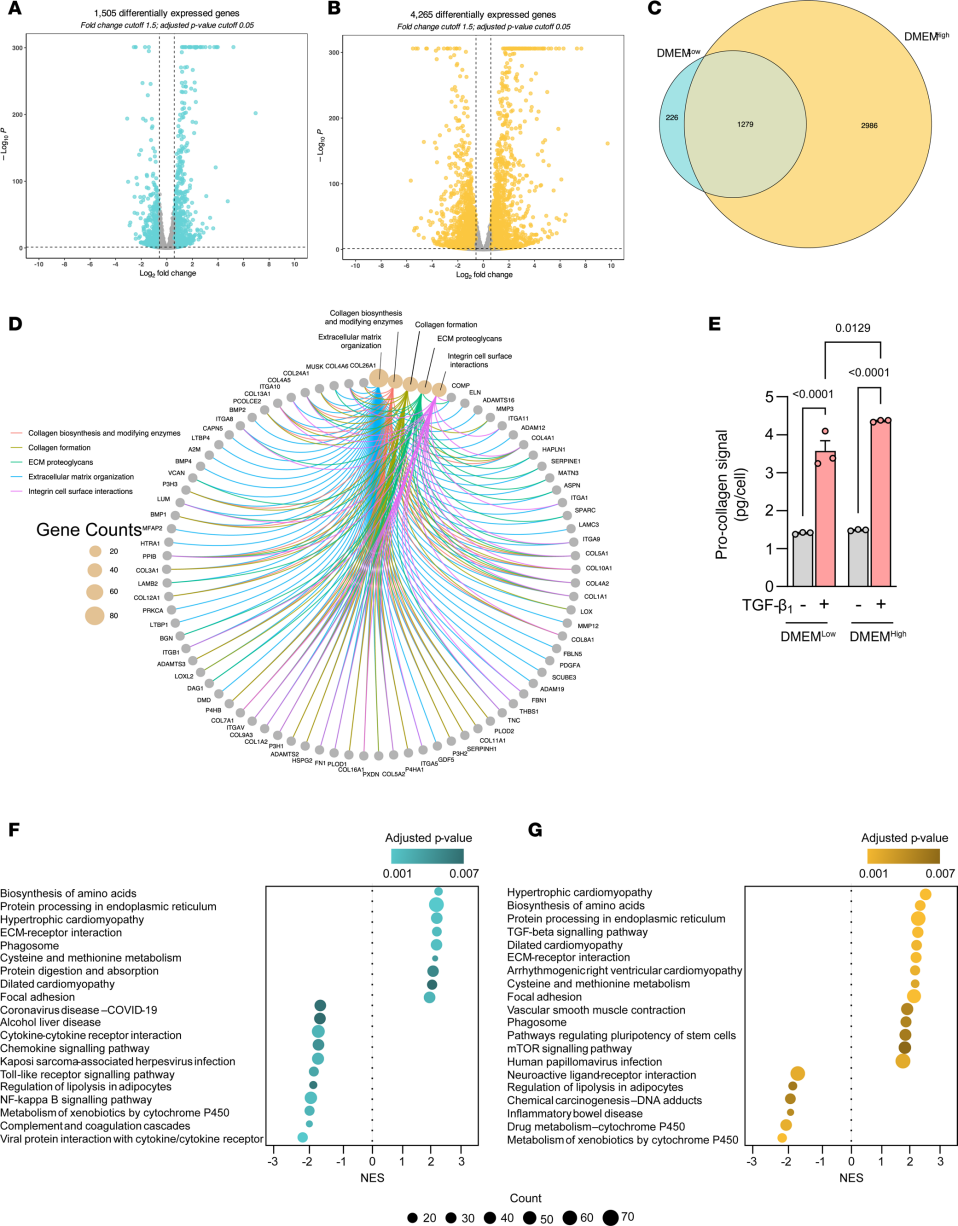
The TGF-β_1_–induced transcriptome is sensitive to media composition. (**A** and **B**) Volcano plots showing differentially expressed genes (DEGs) in (**A**) DMEM^lo^ and (**B**) DMEM^hi^ of pHLFs stimulated with TGF-β_1_ (1 ng/mL) for 24 hours with cutoff values *q* ≤ 0.05 and fold-change ± 1.5. (*n* = 3.) (**C**) Overlap diagram of DEGs between DMEM^lo^ and DMEM^hi^. (**D**) Network plot of enriched Reactome terms of DEGs shared between DMEM^lo^ and DMEM^hi^. (**E**) Supernatants collected from pHLFs stimulated with TGF-β_1_ (1 ng/mL) for 48 hours and hydroxyproline quantified using HPLC (*n* = 3), representative of 5 independent experiments. (**F** and **G**) Dot plots showing top 20 enriched KEGG terms in (**F**) DMEM^lo^ and (**G**) DMEM^hi^. Data are presented as mean ± SD and differences evaluated between groups with 2-way ANOVA with Tukey’s multiple-comparison testing. NES, normalized enrichment score.

Differential gene expression analysis revealed substantial differences between the total number of significantly modulated genes and the magnitude of the fold-changes induced by TGF-β_1_, with DMEM^hi^ leading to nearly 3-fold more differentially expressed genes (DEGs) than DMEM^lo^ (Figure 1, A–C). The high number of unique DEGs in DMEM^hi^ (2,986 DEGs) led us to examine metabolic pathway expression to determine if there was any transcriptional evidence of the influence of high carbon and nitrogen sources available in this media. Kyoto Encyclopedia of Genes and Genomes (KEGG) pathway analysis indeed showed central carbon metabolism as a top hit (*P* = 0.0024), which included genes involved in glycolysis, such as lactate dehydrogenase A and pyruvate dehydrogenase E1 subunit β (Supplemental Figure 1; supplemental material available online with this article; https://doi.org/10.1172/jci.insight.178453DS1). We next wanted to ensure that regardless of the media, the central processes involved in TGF-β_1_ signaling (i.e., ECM remodeling) were retained and significantly modulated. Reactome analysis on these 1,279 shared genes was used to determine these processes, and this revealed terms such as collagen biosynthesis, fibril formation and assembly, ECM formation, and tRNA aminoacylation (the latter potentially to meet the enhanced protein synthetic demands of TGF-β_1_–activated hypersynthetic fibroblasts) (Figure 1D). The ability of both media formulations to support a robust TGF-β_1_ pro-collagen response at the protein level was verified by assessing hydroxyproline levels in cell supernatants, with DMEM^hi^-cultured cells showing a slightly higher response (Figure 1E).

We next performed gene set enrichment analysis (GSEA) for both media formulations shown in Figure 1, A and B. This analysis revealed that almost half of the top 20 gene sets were unique to the media used (Figure 1, F and G). Interestingly, this analysis identified the biosynthesis of amino acids as a top TGF-β_1_–dependent module in both media conditions, indicating that even with markedly higher nutrient sources present in DMEM^hi^, this pathway was highly modulated by TGF-β_1_ in both nutrient environments.

### TGF-β_1_–modulated intracellular amino acid pools influence metabolic vulnerabilities.

**Figure 2:**
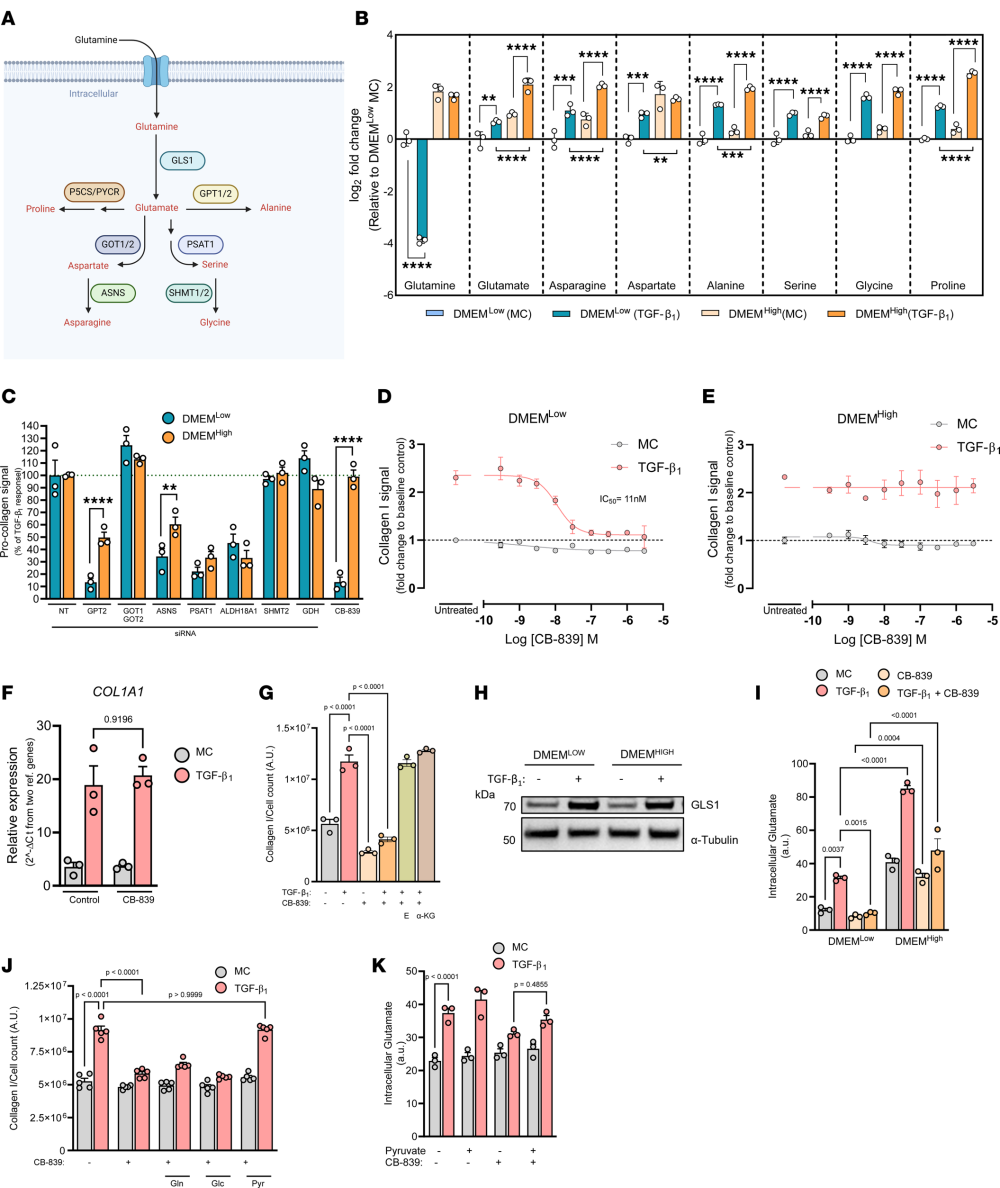
TGF-β_1_–modulated intracellular amino acid pools influence metabolic vulnerabilities. (**A**) Glutaminolysis schematic. GOT, glutamic-oxaloacetic transaminase; ASNS, asparagine synthetase; PSAT1, phosphoserine aminotransferase 1; SHMT1/2, serine hydroxymethyltransferase 1/2. (**B**) Intracellular levels of amino acids in DMEM^lo^ or DMEM^hi^ stimulated with TGF-β_1_ (1 ng/mL) for 48 hours (*n* = 3). (**C**) Supernatants collected from pHLFs stimulated with TGF-β_1_ following transfection with nontargeting siRNA for control and CB-839–treated groups (1 μM) or targeted siRNA and hydroxyproline quantified (*n* = 3). (**D** and **E**) pHLFs were grown in DMEM^lo^ or DMEM^hi^ and preincubated with increasing concentrations of CB-839 before TGF-β_1_ stimulation and collagen I deposition assessed by macromolecular crowding assay. Data are expressed as collagen I signal as fold-change of media control (0.1% DMSO). (**F**) *COL1A1* mRNA levels 24 hours after TGF-β_1_ stimulation from pHLFs growing in DMEM^lo^ and 1 μM CB-839. (**G**) Collagen deposition quantified 48 hours after TGF-β_1_ stimulation from pHLFs growing in DMEM^lo^ and 1 μM CB-839 with supplementation of dimethylated forms of glutamate (E, 4 mM) and α-ketoglutarate (4 mM). (**H**) Immunoblot of intracellular protein lysates from pHLFs 24 hours following TGF-β_1_ stimulation grown in DMEM^lo^ or DMEM^hi^. (**I**) pHLFs were grown in DMEM^lo^ or DMEM^hi^ and stimulated with TGF-β_1_ and CB-839 for 48 hours and glutamate measured by HPLC (*n* = 3). (**J**) pHLFs were grown in DMEM^lo^ supplemented with glutamine (1.3 mM), glucose (20 mM), or pyruvate (1 mM) before preincubation with 1 μM CB-839 before TGF-β_1_ stimulation and collagen I deposition assayed by macromolecular crowding assay. (**K**) Intracellular glutamate levels in pHLFs grown in DMEM^lo^ supplemented with pyruvate (1 mM) and preincubation with CB-839 for 1 hour before TGF-β_1_ stimulation and quantification achieved using HPLC (*n* = 3). Data are presented as mean ± SD. Two-way ANOVA with Tukey’s multiple-comparison testing. **P < 0.01, ***P < 0.001, ****P < 0.0001.

Amino acid biosynthesis is known to be highly dependent on the availability of glutamine, which provides transferrable amino groups to α-ketoacids after its conversion to glutamate (Figure 2A). Given that the glutamine concentration is approximately 3-fold higher in DMEM^hi^ (2 mM versus 0.7 mM in DMEM^lo^), we measured the intracellular levels of glutamate-dependent amino acids following TGF-β_1_ stimulation. We found that TGF-β_1_ decreased intracellular glutamine levels in DMEM^lo^ by more than 90%, to levels barely above the limit of detection by 48 hours; this was in stark contrast with the high levels of intracellular glutamine measured in DMEM^hi^ at baseline and in response to TGF-β_1_ (Figure 2B). We questioned whether this marked decrease in intracellular glutamine levels in TGF-β_1_–treated cells in DMEM^lo^ was due to a depletion of extracellular glutamine levels over the 48-hour culture period. However, we found that extracellular glutamine levels had dropped only by close to 50% (377 μM) at this time point (Supplemental Figure 2A). TGF-β_1_ increased the intracellular levels of all other amino acids measured, and this effect was more pronounced in DMEM^hi^ (except for aspartate levels, which were unchanged by TGF-β_1_). Since glutamate, asparagine, aspartate, alanine, and proline are not added to these DMEM formulations, the increased intracellular levels suggest increased amino acid biosynthesis induced in response to TGF-β_1_ rather than increased uptake from the media. In support of this, we found that TGF-β_1_–induced levels of serine and glycine, which are both present in DMEM, were similar for DMEM^lo^ and DMEM^hi^. Intracellular levels of glutamate in unstimulated cells cultured in DMEM^hi^ were higher than the glutamate levels in TGF-β_1_–treated cells cultured in DMEM^lo^. This might indicate an enhanced capacity of fibroblasts in DMEM^hi^ to synthesize nonessential amino acids owing to a larger pool of available intracellular glutamate.

We next investigated whether the enzymes involved in amino acid biosynthesis were conditionally essential depending on media composition for TGF-β_1_–induced collagen synthesis. At the time of this study, GLS1 was the only enzyme with a pharmacological inhibitor with high selectivity and nanomolar potency, CB-839 (20). We used this compound to address the role of GLS1, and we employed silencing RNA–mediated (siRNA-mediated) gene knockdown for all other enzymes. We opted to silence both the cytoplasmic and mitochondrial forms of GOT, *GOT1* and *GOT2*, respectively, since both can generate aspartate and may therefore compensate for one another (Supplemental Figure 2B). These studies revealed GPT2, ASNS, and GLS1 as being more critical enzymes to enable fibroblasts to mount a TGF-β_1_–induced collagen response in DMEM^lo^ than in DMEM^hi^ (Figure 2C). We further found that *PSAT1* and *ALDH18A1* (gene product is P5CS) were crucial for TGF-β_1_–induced collagen levels regardless of DMEM formulation, which is in line with previous work highlighting the important role of serine and proline biosynthetic pathways in TGF-β_1_–induced collagen synthesis (21, 22). We defined the mechanism of action of GPT2, ASNS, and ALDH18A1 to the absence in DMEM of alanine, asparagine, and proline, respectively, as introduction to the growth media of each respective amino acid completely rescued TGF-β_1_–induced collagen levels in DMEM^lo^ (Supplemental Figure 2, C–H). It is therefore plausible that the greater stores of intracellular alanine and asparagine of fibroblasts in DMEM^hi^ may be attributable for the differences in effect of collagen production between the 2 media observed for siGPT2 and siASNS, respectively. Interestingly, we observed that silencing of *GDH* did not affect TGF-β_1_–induced collagen production in media deprived of glutamate, the amino acid GDH can generate. Instead, we found that siGDH-treated cells had significantly higher levels of intracellular glutamate, indicating a potential compensation by GLS1 or a glutamate-consuming function of GDH in TGF-β_1_–stimulated conditions (Supplemental Figure 2I).

Unlike siGPT2, siASNS, or siALDH18A1 treatment, which reduced TGF-β_1_–induced collagen levels in both DMEM formulations, inhibition of GLS1 completely prevented TGF-β_1_–induced collagen synthesis in DMEM^lo^ (IC_50_= 11 nM) but failed to produce any effect in DMEM^hi^ (Figure 2, D and E). We found this effect to be posttranscriptional, as TGF-β_1_–induced collagen type I alpha 1 chain mRNA levels were unaffected by CB-839 treatment (Figure 2F). GLS1 is the rate-limiting enzyme for glutaminolysis, which produces glutamate for subsequent α-ketoglutarate generation into the tricarboxylic acid (TCA) cycle. We examined this mechanism of action by supplementing fibroblasts with a cell-permeable form of either glutamate or α-ketoglutarate where both individually restored the TGF-β_1_–induced collagen response under GLS1-inhibited conditions (Figure 2G). Two additional GLS1 inhibitors, bis-2-(5-phenylacetamido-1,3,4-thiadiazol-2-yl)ethyl sulfide (BPTES) and Compound 968, effectively abrogated the TGF-β_1_–induced collagen response in DMEM^lo^ but failed to have an effect in DMEM^hi^ (Supplemental Figure 2, J–L). Using an siRNA-mediated knockdown approach further supported the role of GLS1 in mediating the TGF-β_1_–induced collagen response (Supplemental Figure 2, M and N). The media difference in impact of CB-839 on the TGF-β_1_–induced collagen response was not the result of differences in GLS1 protein abundance in the respective media formulations (Figure 2H). Moreover, intracellular TGF-β_1_–stimulated glutamate levels were GLS1 dependent in both media, but cells cultured in DMEM^hi^ retained a much higher abundance of glutamate following CB-839 exposure (Figure 2I). We therefore explored the possibility that the high levels of glutamine present in DMEM^hi^ may allow for a higher glutamate pool, which is sufficient for cells to mount a full TGF-β_1_–induced collagen response without GLS1 activity. However, when examining the cause for this difference by individually adjusting the concentrations of the 3 components that differ between the DMEM formulations (glucose, pyruvate, and glutamine) in DMEM^lo^ to the levels present in DMEM^hi^, we found that it was pyruvate and not glutamine (as expected) which was responsible for restoring the TGF-β_1_–induced collagen response in GLS1-inhibited cells cultured in DMEM^lo^ (Figure 2J). These observations suggest that pyruvate confers resistance to GLS1 inhibition in DMEM^hi^, an effect that was observed from 100 μM (physiological human blood concentration) onward by addition of pyruvate to DMEM^lo^-cultured cells (Supplemental Figure 2O). Furthermore, intracellular glutamate levels were not significantly increased by the presence of pyruvate and were below the levels measured in DMEM^hi^ (Figure 2K). To further explore these findings in fibroblasts derived from patients with IPF, we investigated the role of GLS1 in IPF lines derived from 2 donors. The data obtained verified that GLS1 is critical for TGF-β_1_–induced collagen production in the absence of extracellular pyruvate (Supplemental Figure 2, P and Q). The data so far suggest that while the high intracellular glutamate levels present in DMEM^hi^ were likely a result of high glutamine availability, the presence of pyruvate overrides the susceptibility of cells to GLS1 inhibition during TGF-β_1_–induced collagen deposition by affecting another mechanism rather than by influencing glutamate synthesis directly.

### Exogenous pyruvate supports the TCA cycle under GLS1 restriction.

**Figure 3:**
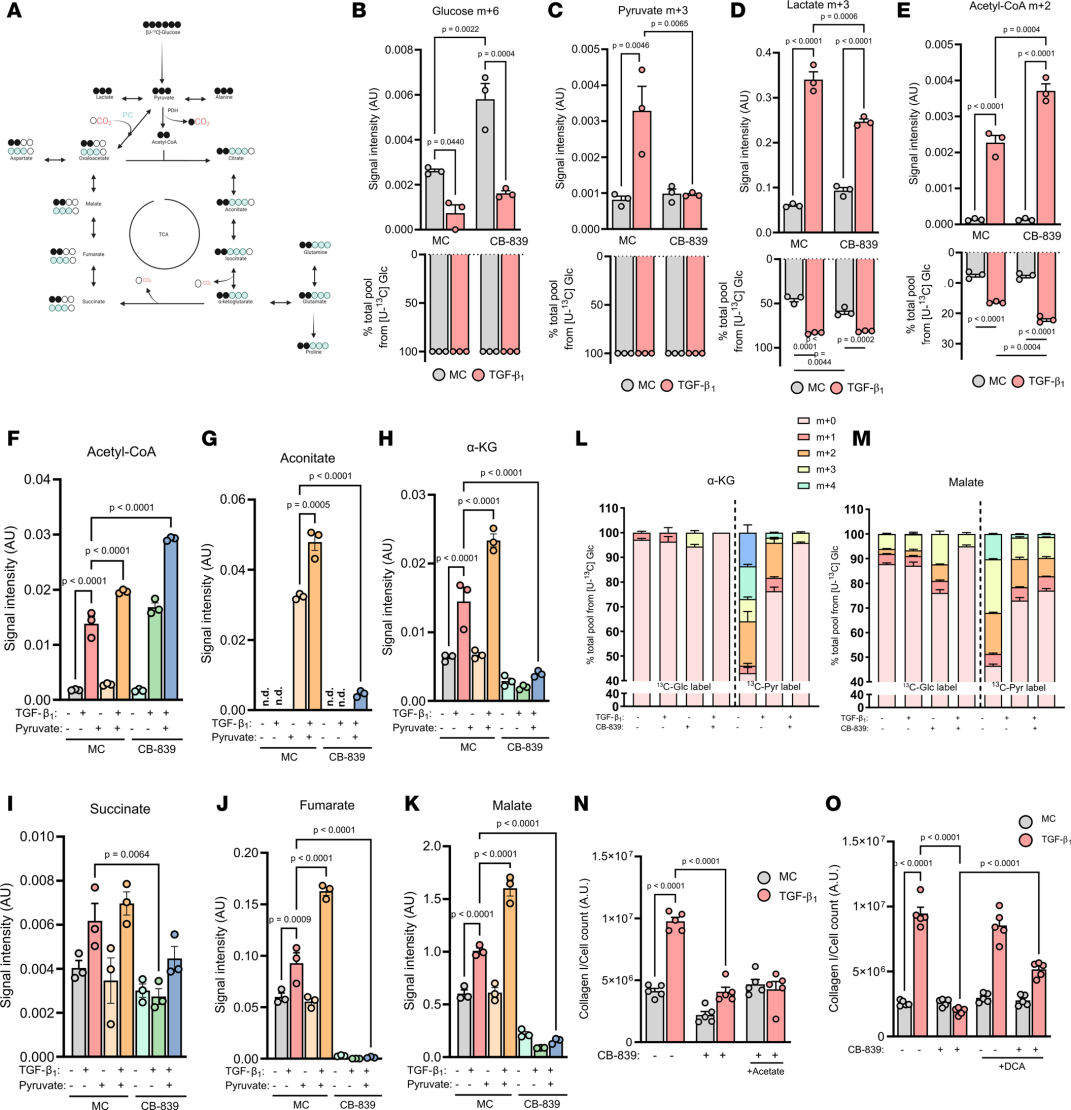
Exogenous pyruvate supports the TCA cycle under GLS1 restriction. (**A**) Schematic showing [U-^13^C]-glucose routing to pyruvate and relevant TCA cycle intermediates and amino acids. (**B**–**E**) Intracellular isotopolog levels and fractional enrichment of specified metabolites in pHLFs grown in DMEM^lo^ supplemented with [U-^13^C]-glucose (5 mM) and preincubated with media control (0.1% DMSO) or 1 μM CB-839 for 1 hour before stimulation with TGF-β_1_ (1 ng/mL) for 48 hours and quantification using LC-MS (*n* = 3). (**F**–**M**) Intracellular levels of specified metabolites or (**L** and **M**) fractional enrichment in pHLFs grown in DMEM^lo^ supplemented with [U-^13^C]-glucose (5 mM) or [U-^13^C]-pyruvate (1 mM) and preincubated with media control (0.1% DMSO) or 1 μM CB-839 for 1 hour before stimulation with TGF-β_1_ (1 ng/mL) for 48 hours and quantification using LC-MS (*n* = 3). (**N** and **O**) Collagen deposition quantified 48 hours after TGF-β_1_ (1 ng/mL) stimulation from pHLFs growing in DMEM^lo^ and 1 μM CB-839 with supplementation of acetate (1 mM) or dichloroacetate (DCA, 10 mM).

Depriving cancer cells of glutamine-derived glutamate via the inhibition of GLS1 is being actively pursued as a therapeutic strategy in the oncology setting and has also been previously explored as a novel antifibrotic approach in preclinical studies (23–25). Pyruvate is the end product of glycolysis and is also a source of cytosolic NAD^+^ through lactate synthesis and a major anaplerotic substrate. The most immediate functional overlap between pyruvate metabolism and GLS1-directed metabolism resides in the TCA cycle (Figure 3A). We therefore sought to further understand the gene-nutrient interaction between GLS1 and pyruvate in DMEM^lo^ to potentially uncover mechanistic overlaps, which may aid in the future translation of therapeutic approaches involving GLS1 antagonists in the setting of fibrosis. We first explored the fate of endogenously produced pyruvate into lactate and acetyl-CoA with fully [^13^C]-labeled glucose under GLS1-restricted conditions using liquid chromatography-mass spectrometry (LC-MS). TGF-β_1_ decreased intracellular glucose levels, but this was likely due to increased glycolytic flux because of a higher pyruvate pool, which was found to be completely m+3 labeled (Figure 3, B and C). Interestingly, CB-839 treatment prevented this increase in glucose-derived pyruvate. We also observed higher glucose levels in control fibroblasts with CB-839 treatment, yet this did not translate to higher pyruvate synthesis. This suggests an inability for cells to effectively metabolize glucose into pyruvate under GLS1-inhibited conditions. Indeed, with CB-839 treatment, we found that even in the presence of 25 mM glucose (the concentration present in DMEM^hi^), which increased intracellular glucose levels by more than 13-fold compared with 5 mM glucose present in DMEM^lo^, pyruvate levels were unchanged (Supplemental Figure 3, A–C). This decrease in pyruvate levels by CB-839 translated into a lower m+3 lactate pool, yet the labeling percentage was unchanged, indicative of the pyruvate contribution to lactate (Figure 3D). We investigated if this decrease in lactate could affect TGF-β_1_–induced collagen levels. We supplemented the media with lactate before CB-839 treatment but found no changes in the diminished collagen response or pyruvate levels (Supplemental Figure 3, D and E). Additionally, we found that with 25 mM glucose (which did not modulate the effectiveness of CB-839 on TGF-β_1_–induced collagen levels) the lactate pool was fully restored, further indicating that lactate generation was not a mechanism by which pyruvate was working to rescue TGF-β_1_–induced collagen production (Supplemental Figure 3C).

We next focused on acetyl-CoA levels and found that the m+2 signal and the label percentage, indicative of pyruvate dehydrogenase (PDH) activity, were significantly increased by TGF-β_1_ in DMEM^lo^ (Figure 3E). Inhibition of GLS1 increased these changes, suggesting that cells adapt to the inhibition of glutaminolysis, a major anaplerotic pathway, by enhancing glucose routing into the TCA cycle via acetyl-CoA generation. We therefore explored the possibility that exogenous pyruvate may support this compensatory mechanism to levels necessary to sustain a full TGF-β_1_–induced collagen response. We found that pyruvate supplementation significantly increased TGF-β_1_–induced acetyl-CoA levels and increased these levels even further under conditions of GLS1 inhibition, indicative of heightened PDH activity (Figure 3F). Even though we did not measure a decrease in acetyl-CoA levels following CB-839 treatment, this result suggested that the overflow of acetyl-CoA may be compensating for a potentially decreased contribution from glutaminolysis to the TCA cycle. Indeed, we found the levels of intermediates of the TCA cycle significantly decreased following GLS1 inhibition (Figure 3, G–K). However, supplementation with pyruvate did not alleviate this depression of the TCA cycle. Interestingly, we were only able to detect aconitate in fibroblasts exposed to extracellular pyruvate (Figure 3G).

To examine whether pyruvate is feeding into the TCA cycle as a carbon substrate, we traced the fate of [^13^C]-labeled pyruvate into α-ketoglutarate and malate. We found that exogenous pyruvate contributed more than glucose-derived pyruvate to the total pools of both of these TCA cycle metabolites in both control and TGF-β_1_ conditions (Figure 3, L and M). GLS1 inhibition completely prevented glucose routing into α-ketoglutarate while exogenous pyruvate could maintain an m+3 pool. GLS1 inhibition also decreased the m+2 and m+3 fractions of glucose-derived malate, an effect that was not observed with supplemented pyruvate. These observations suggest that glucose-derived pyruvate had lower flux into the TCA cycle than exogenous pyruvate following GLS1 inhibition and that although the total numbers of TCA cycle intermediates were significantly decreased by CB-839 (Figure 3, G–K), there existed an active route for pyruvate to sustain both PDH and pyruvate carboxylase (PC) activity. To test this further, we provided fibroblasts acetate as a means of elevating acetyl-CoA levels. However, we found this did not alleviate the reduction in TGF-β_1_–induced collagen levels by CB-839 (Figure 3N). Given that acetate conversion to acetyl-CoA is an ATP-dependent process and that the TCA cycle is severely depressed by GLS1 inhibition, we explored whether increasing PDH activity, which uses NAD^+^, via dichloroacetate (DCA), a potent inhibitor of all PDH kinases, would rescue the TGF-β_1_–induced collagen response. DCA treatment in CB-839–treated cells significantly increased TGF-β_1_–induced collagen levels to roughly 57% of the TGF-β_1_ response, compared with 22% without DCA treatment (Figure 3O). These results suggest that at least some of the rescue mechanism(s) of pyruvate on CB-839-inhibited TGF-β_1_–induced collagen levels resides in propping up the TCA cycle and that enhancing pyruvate flux into the TCA cycle can modulate CB-839 sensitivity.

### Exogenous pyruvate supports amino acid biosynthesis under GLS1 restriction.

**Figure 4:**
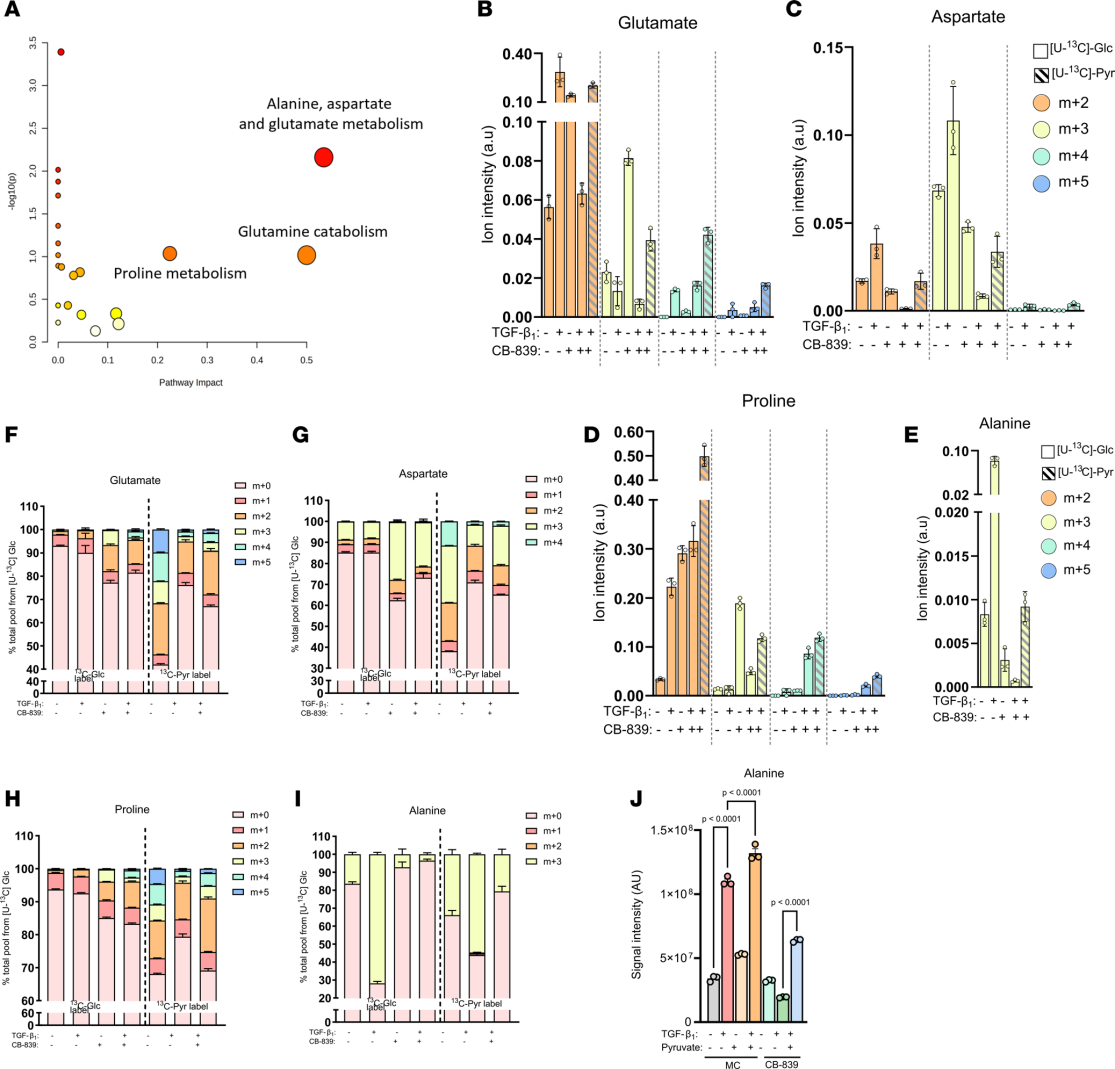
Exogenous pyruvate supports amino acid biosynthesis under GLS1 restriction. (**A**) Pathway analysis using MetaboAnalyst 5.0 of metabolites with high [U-^13^C]-pyruvate enrichment in 1 μM CB-839–treated and TGF-β_1_–stimulated (1 ng/mL) pHLFs following 48 hours. (**B**–**J**) Intracellular isotopolog levels, (**F**–**I**) fractional enrichment, and (**J**) total abundance of specified metabolites in pHLFs grown in DMEM^lo^ supplemented with [U-^13^C]-glucose (5 mM) or [U-^13^C]-pyruvate (1 mM) and preincubated with media control (0.1% DMSO) or 1 μM CB-839 for 1 hour before stimulation with TGF-β_1_ (1 ng/mL) for 48 hours and quantification using LC-MS (*n* = 3).

The partial rescuing effect of DCA on CB-839–decreased collagen production suggested that exogenous pyruvate may be facilitating a complete rescue by a mechanism(s) beyond anaplerosis. To examine this further, we performed an untargeted analysis using [U-^13^C]-pyruvate under GLS1 restriction and under TGF-β_1_ stimulation and extended our labeling time to 48 hours to fully encompass all potential metabolites that may be mediating the rescue functions of pyruvate. This analysis identified 34 potential metabolites with high ^13^C enrichment and 27 of which were increased by pyruvate supplementation (Supplemental Data 1). Pathway analysis of these 27 metabolites revealed alanine, aspartate, and glutamate metabolism as having the top pathway impact score, with glutamine catabolism and proline metabolism being second and third, respectively (Figure 4A). Although we did detect malate and other TCA cycle intermediates in our untargeted analysis, the list of 27 metabolites was dominated by amino acids and acetylated amino acids. We therefore referenced our tracing data and analyzed the peaks for glutamate, aspartate, proline, and alanine. To enable comparisons between the labels, we first examined the isotopolog signal intensities to define total amounts contributed by either glucose or supplemented pyruvate following TGF-β_1_ stimulation and GLS1 inhibition. This analysis revealed that exogenous pyruvate increased (compared with glucose) the total amounts of glutamate, aspartate, and proline derived from both PDH (m+2) and PC (m+3) under conditions of GLS1 inhibition and TGF-β_1_ stimulation (Figure 4, B–D). Pyruvate was also superior at generating alanine through GPT2 (indicated by the m+3 fraction) under these conditions to glucose-derived pyruvate (Figure 4E). Examining total percentage contributions of each label, we found that exogenous pyruvate sustained a greater fraction of the glutamate, aspartate, and proline pools than glucose when GLS1 was inhibited (Figure 4, F–H). Alanine was unique in that over 70% was labeled from glucose by TGF-β_1_, and this was reduced to less than 5% by GLS1 inhibition (Figure 4I). This strong decrease in carbon flux was likely due to limiting availability of pyruvate (the carbon backbone of alanine) and glutamate (the amino group provider for alanine). GPT2 uses both of these substrates to generate alanine, and pyruvate flux through this enzyme was substantially increased by supplementation with pyruvate under CB-839– and TGF-β_1_–treated conditions, supporting roughly 20% of the total alanine pool (Figure 4I). Out of these 4 amino acids, only alanine was significantly increased 48 hours into TGF-β_1_ signaling under GLS1 restriction with pyruvate present in the media (Figure 4J and Supplemental Figure 3, F–H). Taken together, these data show that TGF-β_1_–stimulated cells have a greater ability to utilize exogenous pyruvate than glucose-derived pyruvate by increasing the isotopolog pool of glutamate, aspartate, proline, and alanine following GLS1 inhibition.

### Pyruvate confers resistance to GLS1 inhibition through GDH-driven glutamate and GPT2-driven alanine biosynthesis.

**Figure 5:**
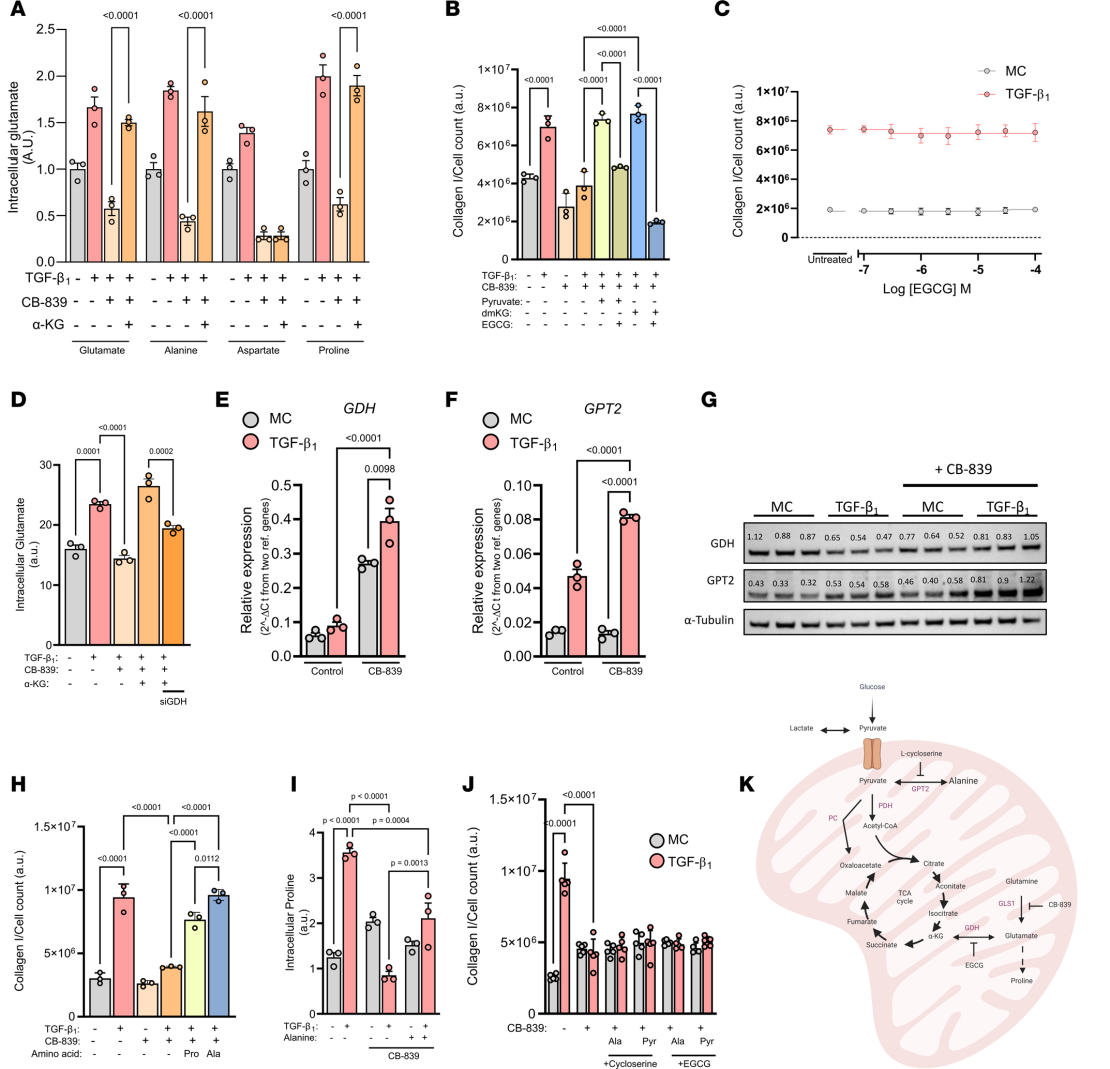
Pyruvate confers resistance to GLS1 inhibition through GDH-driven glutamate and GPT2-driven alanine biosynthesis. (**A**) Intracellular amino acid levels of pHLFs supplemented with dimethyl-α-ketoglutarate (4 mM) with 1 μM CB-839 and TGF-β_1_ (1 ng/mL) for 48 hours. (**B**) pHLFs were supplemented with dimethyl-α-ketoglutarate (4 mM) or pyruvate (1 mM) and preincubated with 1 μM CB-839 or 50 μM EGCG before stimulation with TGF-β_1_ (1 ng/mL) for 48 hours, and collagen I deposition was assessed by macromolecular crowding assay. (**C**) pHLFs were preincubated with increasing concentrations of EGCG for 1 hour before being stimulated with TGF-β_1_ (1 ng/mL) for 48 hours, and collagen I deposition was assessed by macromolecular crowding assay. (**D**) pHLFs were transfected with nontargeting (NT) siRNA or siRNA targeting GDH, cells were preincubated with 1 μM CB-839 and dimethyl-α-ketoglutarate (4 mM) before stimulation with TGF-β_1_ (1 ng/mL), and intracellular glutamate levels were quantified by HPLC after 48 hours. (**E** and **F**) pHLFs were treated with 1 μM CB-839 for 1 hour prior to TGF-β_1_ (1 ng/mL) stimulation for 24 hours, and RNA was quantified using quantitative PCR. (**G**) Immunoblot from pHLFs 24 hours following TGF-β_1_ stimulation (1 ng/mL) and 1 μM CB-839. (**H**) pHLFs were treated with 1 μM CB-839 for 1 hour supplemented with alanine or proline (500 μM) prior to TGF-β_1_ (1 ng/mL) stimulation for 48 hours, and collagen I deposition was assessed by macromolecular crowding assay. (**I**) pHLFs were supplemented with alanine (500 μM) before stimulation with TGF-β_1_ (1 ng/mL) and CB-839 (1 μM) for 48 hours, with intracellular proline measured by HPLC (*n* = 3). (**J**) pHLFs were treated as in **B** with alanine (500 μM) or pyruvate (1 mM) supplementation prior to a 1-hour preincubation with 20 μM l-cycloserine or 50 μM EGCG before stimulation with TGF-β_1_ (1 ng/mL) for 48 hours, and collagen I deposition was assessed by macromolecular crowding assay. (**K**) Schematic showing pyruvate and glutamine routing into the TCA cycle. Data are presented as mean ± SD, and differences were evaluated between groups with 2-way ANOVA with Tukey’s multiple-comparison testing.

Our data so far strongly indicated that pyruvate was routing through the TCA cycle into glutamate to then bypass GLS1 inhibition. Our earlier results showed that both glutamate and α-ketoglutarate were capable of fully rescuing TGF-β_1_–induced collagen levels with CB-839 treatment (Figure 2G). Given that α-ketoglutarate and glutamate are part of many bidirectional metabolic reactions, we questioned whether the cell-permeable form of α-ketoglutarate was mediating a rescue through regeneration of glutamate levels following GLS1 inhibition. Indeed, we found that the intracellular levels of not only glutamate but also of alanine and proline were completely restored to TGF-β_1_–induced levels by α-ketoglutarate supplementation (Figure 5A). We found that α-ketoglutarate supplementation did not regenerate aspartate levels, which together with our silencing data of GOT1/GOT2 (Figure 2C) suggested a dispensable role for aspartate biosynthesis during TGF-β_1_–induced collagen production.

Glutamate synthesis from α-ketoglutarate is a reversible reaction largely mediated by aminotransferases, including GPT2 or GOT2, and GDH (26). The former requires the degradation of an amino acid, and since GLS1 inhibition decreased amino acid levels, this seemed unlikely to be the chosen pathway for glutamate synthesis following pyruvate supplementation. We therefore decided to use the GDH inhibitor epigallocatechin gallate (EGCG), to determine if pyruvate and α-ketoglutarate were still capable of rescuing TGF-β_1_–induced collagen synthesis following CB-839 treatment. We found that inhibition of GDH, which is dispensable during TGF-β_1_–mediated collagen synthesis (Figure 2C), prevented both pyruvate and α-ketoglutarate from rescuing collagen production under GLS1-inhibited conditions (Figure 5, B and C). We observed that GDH inhibition decreased TGF-β_1_–induced collagen levels to a much lower extent with α-ketoglutarate than with pyruvate, suggesting that pyruvate primarily rescues CB-839 through amino acid synthesis rather than by fueling the TCA cycle. We next measured intracellular glutamate levels under these conditions and found that silencing of GDH significantly decreased the regenerated levels of glutamate following α-ketoglutarate supplementation (Figure 5D). These data showcase the resilience of the glutaminolytic axis and highlight that dual targeting of GLS1-derived glutamate and GDH-derived glutamate is required to efficiently prevent TGF-β_1_–induced collagen synthesis. In further support of this, we found that inhibition of GLS1 led to an increase in both GPT2 and GDH mRNA levels and protein abundance, potentially indicating a physiologically relevant compensation mechanism (Figure 5, E–G).

We next individually introduced alanine and proline to examine whether CB-839–inhibited TGF-β_1_–induced collagen levels were restored. We found that proline induced a partial rescue while alanine was able to fully restore TGF-β_1_–induced collagen levels decreased by GLS1 inhibition (Figure 5H). We therefore questioned how alanine might facilitate a complete rescue in GLS1-restricted conditions if the levels of proline (critical for collagen synthesis) were dependent on GLS1-derived glutamate (Supplemental Figure 3H). We found that supplementation with alanine in CB-839–treated cells significantly increased TGF-β_1_–stimulated proline levels, indicating that alanine was capable of substituting for glutamate when GLS1 activity was inhibited (Figure 5I). This would suggest a reversible function for GPT2, which degrades alanine to aminate α-ketoglutarate for glutamate generation. In support of this, we found that inhibiting GPT2 with l-cycloserine completely prevented alanine from rescuing GLS1 inhibition (Figure 5J). Moreover, l-cycloserine also prevented pyruvate from rescuing GLS1 inhibition, which highlights alanine biosynthesis as a major function of the pyruvate rescue (Figure 5, J and K).

### GLS1 and GDH dual inhibition prevents TGF-β_1_–induced collagen secretion in precision-cut lung slices.

**Figure 6:**
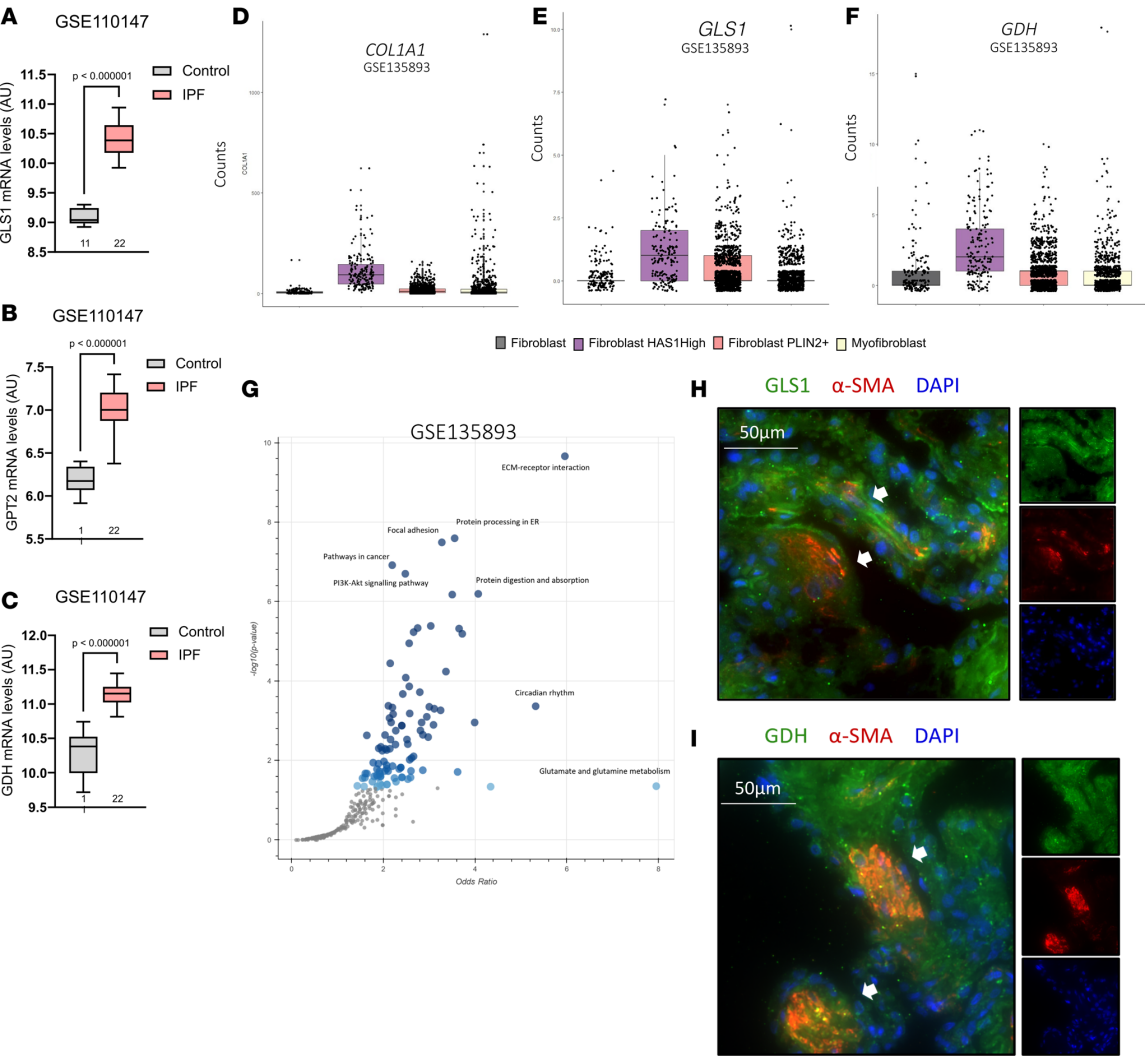
GLS1 and GDH are expressed by activated fibroblasts in IPF. (**A**–**C**) mRNA expression of indicated genes in GEO GSE110147 between a control lung and IPF lung. Patient numbers are indicated. Box plots show the interquartile range, median (line), and minimum and maximum (whiskers). (**D**–**F**) mRNA expression of indicated genes in GEO GSE135893 comparing 4 fibroblast subpopulations found in IPF lung tissue. (**G**) Pathway analysis (KEGG) of 1,244 upregulated genes (*P* < 0.05) derived from comparing control lung myofibroblasts and IPF lung myofibroblasts. (**H** and **I**) IPF lung tissue double-immunofluorescence staining for (**H**) GLS1 and α–smooth muscle actin and (**I**) GDH and α–smooth muscle actin. Arrows indicate myofibroblast-concentrated regions in representative images shown of 3 patients with IPF.

From a therapeutic perspective, our data highlight 2 potential mechanisms by which a GLS1 inhibitor strategy alone may be hampered in the setting of pulmonary fibrosis: (a) through pyruvate-sustained glutamate levels via GDH and (b) through upregulation of GDH and GPT2 following GLS1 inhibition. With this in mind, we assessed the expression of the genes encoding these enzymes in the setting of IPF in publicly available RNA-Seq datasets and found that all 3 genes were significantly elevated in IPF lung tissue compared with control lung tissue (Figure 6, A–C) (27). To determine if these genes are upregulated in pathological fibroblasts, we next analyzed an IPF lung single-cell RNA-Seq dataset, which was the first to identify 4 pathogenic fibroblast subpopulations enriched in IPF, including a subpopulation of IPF fibroblasts characterized by high expression of hyaluronan synthase 1 and *COL1A1* (Figure 6D) (28). Interestingly, *GPT2* expression was low and did not pass the significance cutoff (*P* ≤ 0.05), yet expression values for both *GLS1* and *GDH* were highest in the *COL1A1*^hi^ subpopulation compared with the other 3 fibroblast groups identified in IPF (Figure 6, E and F). We next analyzed all the DEGs in IPF myofibroblasts compared with controls, which identified 1,244 genes significantly increased in IPF myofibroblasts. Encouragingly, subsequent pathway analysis revealed glutamate and glutamine metabolism as a top enriched pathway, which consisted of just 2 genes: *GLS1* and *GDH* (Figure 6G). Other significant pathways identified were in line with expectations for a fibrotic disease and included, for example, ECM-receptor interaction and protein processing. Furthermore, the glutamate-consuming and glutamine-synthesizing enzyme glutamine synthase (*GLUL*) was among the top downregulated genes in IPF myofibroblasts (60% reduction, *P* = 7.2 × 10^–13^).

We next sought to verify these findings at the protein level in IPF lung tissue using immunofluorescence to observe potential colocalization of GLS1 or GDH with the myofibroblast marker, α–smooth muscle actin (α-SMA). These studies showed that both GLS1 and GDH were extensively expressed by IPF lung tissue and, crucially, expressed by α-SMA^+^ myofibroblasts (Figure 6, H and I). Moreover, we found that the GDH protein signal was higher in myofibroblasts than the overlying epithelium (Figure 6I). Confidence in antigen specificity was addressed with both IgG and unstained controls, which showed very low fluorescence signals.

**Figure 7:**
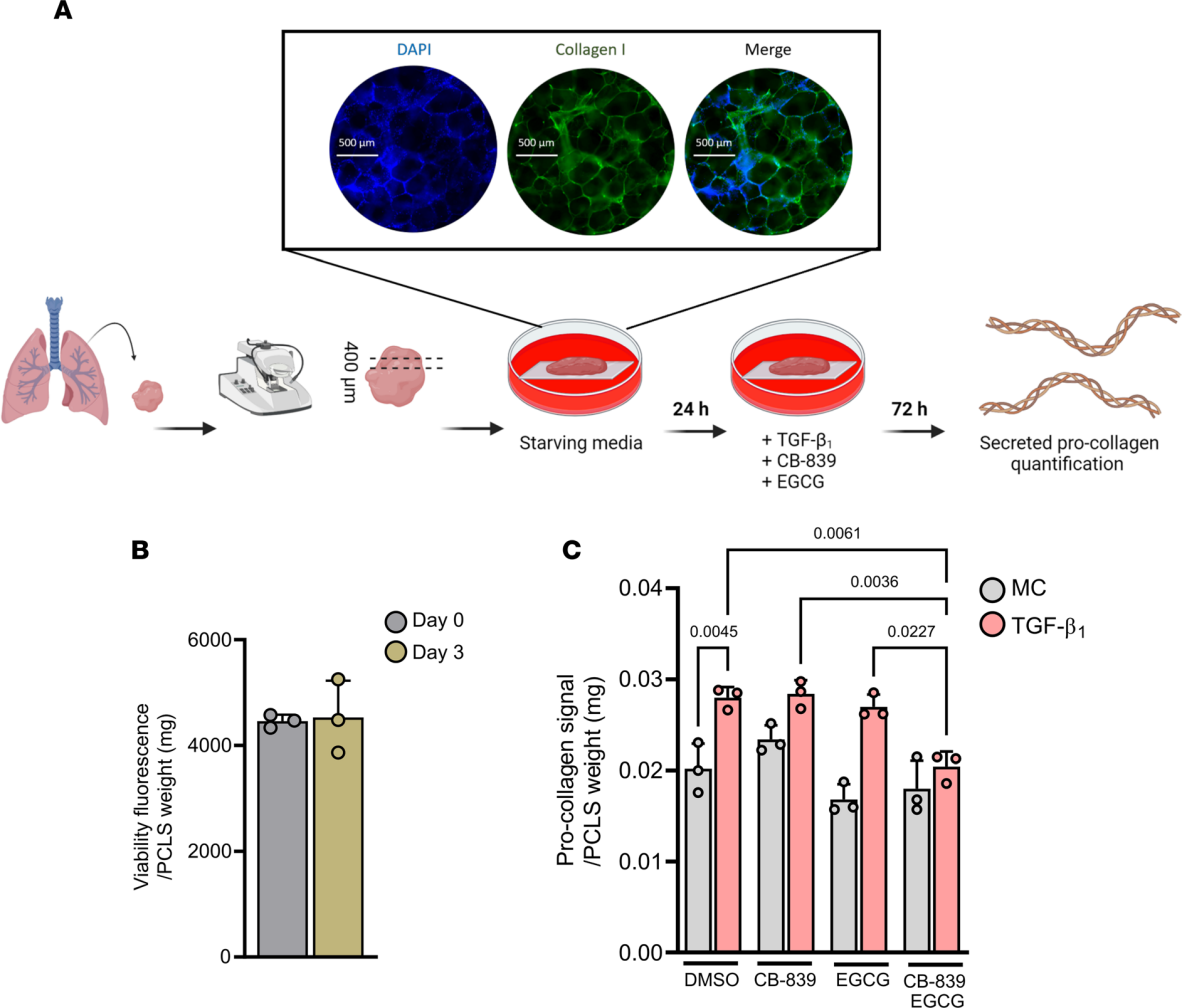
Dual inhibition of GLS1 and GDH prevents TGF-β_1_–induced collagen secretion in precision-cut ex vivo lung slices. (**A**) Schematic illustrating the experimental process for precision-cut ex vivo lung tissue slices (PCLS) and showing immunohistochemical visualization of cell nuclei (DAPI) and tissue structure (collagen I). (**B**) Tissue slice viability quantified via reduction of resazurin (560/590 nm excitation/emission). (**C**) PCLS were treated with 1 μM CB-839 or 50 μM EGCG before stimulation with TGF-β_1_ (10 ng/mL) for 72 hours and soluble pro-collagen quantification via reverse-phase HPLC detection of hydroxyproline. Data are presented as mean ± SD, and differences are evaluated between groups with 2-way ANOVA with Tukey’s multiple-comparison testing.

**Figure 8:**
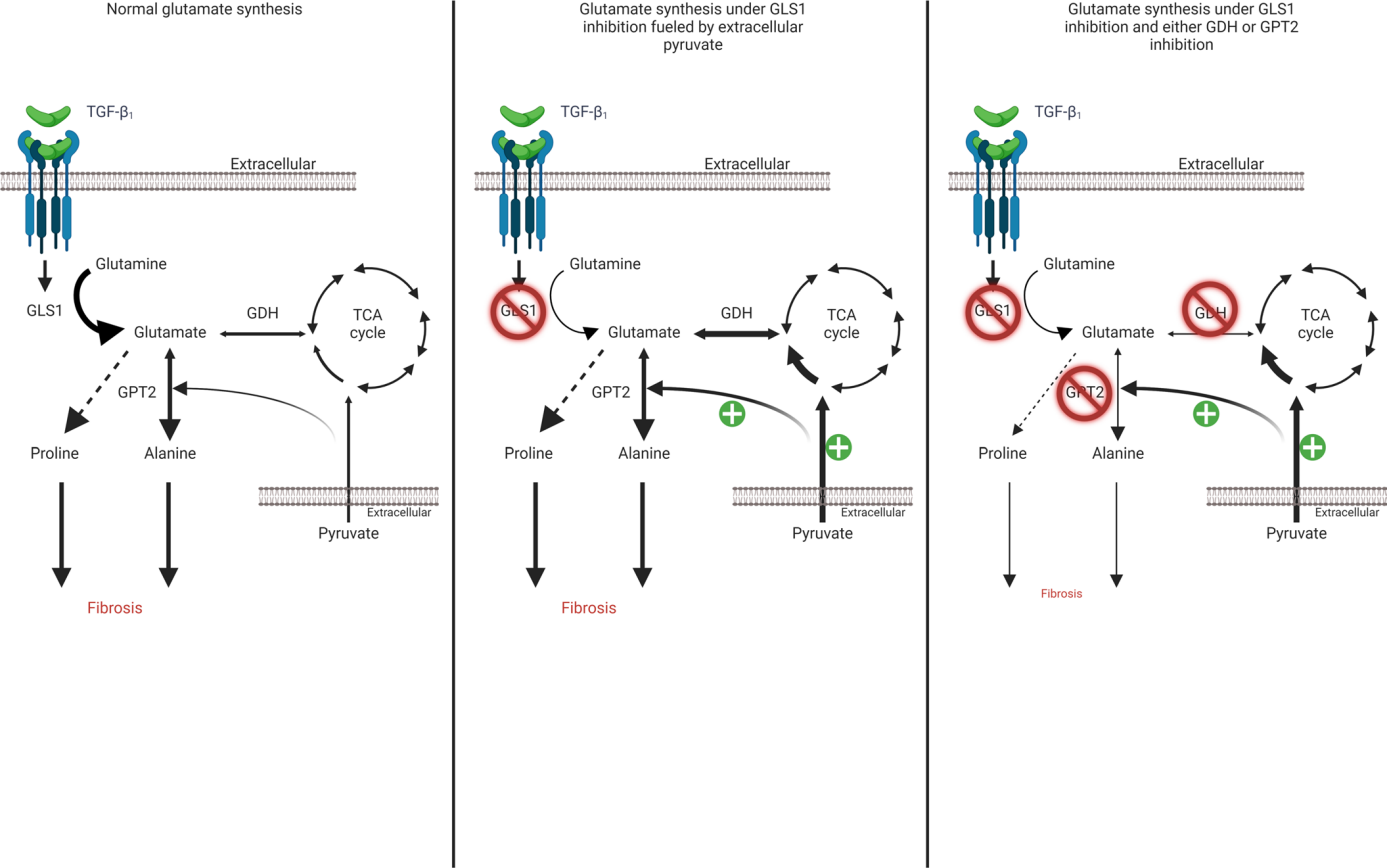
Proposed mechanism by which pyruvate routing under GLS1-inhibited conditions sustains fibrogenesis through GDH and GPT2. TGF-β_1_ increases GLS1 expression, which leads to a conversion of intracellular glutamine levels to glutamate, which supports alanine and proline biosynthesis, necessary for a TGF-β_1_–induced collagen response. Following GLS1 restriction, glutamate, alanine, and proline levels decrease, which are capable of being sustained by exogenous pyruvate, which supplies glutamate through TCA cycle routing. Preventing this routing by inhibition of GDH or preventing alanine biosynthesis by inhibition of GPT2 are 2 strategies to effectively limit the TGF-β_1_–induced collagen response under GLS1 restriction. TGF-β_1_, transforming growth factor β_1_; GLS1, glutaminase 1; GDH, glutamate dehydrogenase; GPT2, glutamic-pyruvic transaminase 2.

So far our data highlighted GDH as a strong contender to inhibit alongside GLS1 to prevent compensatory mechanisms that allow myofibroblasts to mount a fibrogenic collagen response. We last wanted to investigate this dual-inhibition strategy in the context of human fibrotic lung disease using live precision-cut lung slices (PCLS) generated from lung biopsies (Figure 7A). We used TGF-β_1_ as the pro-fibrotic stimulus and cultured these PCLS for 72 hours in pyruvate-replete DMEM prior to quantification of soluble pro-collagen in culture supernatants. We verified that tissue viability was maintained throughout the experimental window via similar reduction efficiencies of resazurin (Figure 7B). Procollagen levels were increased following 72 hours of TGF-β_1_ stimulation and were insensitive to singular GLS1 or GDH inhibition (Figure 7C). Crucially, combination treatment with both inhibitors was successful in preventing the enhanced pro-collagen response induced by TGF-β_1_. These PCLS data extend the potential relevance of our observations beyond the limitations of a monoculture system and further support a therapeutic strategy based on dual targeting of the glutamate-synthesizing enzymes GLS1 and GDH to exploit the metabolic vulnerabilities of hypersecretory pathogenic fibroblast subpopulations as a potentially novel antifibrotic approach (Figure 8).

## Discussion

Fibroblasts play a central role in the turnover of the ECM in homeostasis and during tissue repair. In fibrosis, this turnover becomes dysregulated and culminates in the progressive accumulation of ECM proteins, primarily collagens, which leads to the obliteration of tissue architecture and eventual organ failure. This pathological process has also been implicated in promoting tumor growth as part of the stromal reaction in cancer (15). In this study, we report that media composition, particularly changes in the extracellular concentrations of glucose, glutamine, and pyruvate, have a major influence on the global TGF-β_1_–regulated transcriptome and intracellular amino acid profile in primary human lung fibroblasts. We further uncover a critical role for pyruvate metabolism in influencing the impact of glutaminolysis inhibition on the TGF-β_1_–induced collagen response. We trace this to a GDH-dependent glutamate and a GPT2-dependent alanine biosynthetic axis. In addition to furthering our fundamental understanding of glycolysis during the TGF-β_1_–induced fibrogenic response, these findings have important implications for the development of novel therapeutic approaches targeting the metabolic vulnerabilities of hypersynthetic fibroblasts.

Media composition greatly affected the global transcriptional response to TGF-β_1_, in terms of the number of genes impacted as well as the magnitude of the response. We elected to compare DEGs for the TGF-β_1_ response and not to perform batch correction for the 2 bulk RNA-Seq datasets performed in the 2 media formulations, since there was no common experimental group to allow standardization. It is also worth commenting that the 2 RNA-Seq datasets were performed using different RNA extraction methods, which may further introduce an unaccounted degree of systemic bias. While we did not individually examine the influence of glucose, glutamine, or pyruvate on the TGF-β_1_ transcriptome, there is evidence that high carbon load (glucose and pyruvate) can affect the cellular epigenome through alteration of chromatin acetylation and methylation sites, which in turn facilitates transcription factor accessibility to a greater number of genes (29, 30). The major acetylation substrate, acetyl-CoA, has recently been described as a key metabolic regulator of the epigenome, and our untargeted metabolomic data presented here identified a large number of acetyl-CoA–conjugated metabolites enriched in the presence of pyruvate. Furthermore, our labeling study showed that pyruvate supplementation greatly increased TGF-β_1_–stimulated acetyl-CoA levels. It is therefore tempting to speculate that the presence of extracellular pyruvate may influence transcriptional responses via an epigenetic modifying mechanism.

In this study we focused on exploring the contribution of media composition in terms of the antifibrotic potential of CB-839, a GLS1 inhibitor currently under investigation in several cancer trials (NCT02071862, NCT02071927, NCT02071888, among others). This compound was found to be effective in halting the TGF-β_1_–induced collagen response in conditions of limited pyruvate or alanine availability and was partially effective in conditions of proline availability or insufficient glycolytic flux leading to pyruvate utilization in the mitochondria. We traced the effects of extracellular pyruvate in mediating resistance to GLS1 inhibition during TGF-β_1_–induced collagen synthesis to its ability to act as a substrate for alanine biosynthesis and, to a lesser extent, proline biosynthesis. Pyruvate concentration is often undefined in conventional cell culture media, and glucose and glutamine are often set at supraphysiological concentrations, making it difficult to compare findings across different studies. We found that supraphysiological levels of glutamine, glucose, and pyruvate led to a higher pool of intracellular alanine, which may explain why other studies using conventional fibroblast media may have underestimated the importance of GPT2 as a critical enzyme for TGF-β_1_–driven collagen production (31, 32). In the cancer setting, GPT2 and GLS1 were identified following a CRISPR screen as critical enzymes for cell viability dependent on extracellular alanine and pyruvate availability, respectively (14). To further confound cross-analysis of relevant fibrosis studies, the quantification of collagen can be performed using various technologies, which measure collagen synthesis at different steps during the biosynthetic process. For example, CB-839 has been reported to show some efficacy in inhibiting intracellular collagen I levels following TGF-β_1_ stimulation in vitro and to partially inhibit total lung collagen levels in experimental lung fibrosis models (23). Our data show that the antifibrotic effect of GLS1 inhibition is highly dependent on the availability of key carbon and nitrogen sources, and the inhibitory effect is enhanced with additional targeting of GPT2 or GDH. The observation that GDH is highly expressed in IPF fibrotic foci supports the rationale of targeting this enzyme in conjunction with GLS1 as a more promising approach. It is also worth highlighting that the inhibitor EGCG is under investigation in a phase I clinical trial to assess safety, pharmacokinetics, and biomarker measurements of drug effect in patients with IPF already receiving background therapy for IPF with either nintedanib or pirfenidone (NCT05195918).

The link between pyruvate and glutamine metabolic pathways has garnered much interest in the oncology setting, where pyruvate has been shown to sustain the CB-839–depleted TCA cycle in proliferating cancer cells (33). Although we did find that the levels of TCA cycle intermediates following TGF-β_1_ stimulation were dependent on GLS1 in fibroblasts, we found a limited impact on these levels following pyruvate supplementation and found that the pyruvate-mediated rescue of CB-839 treatment on enhanced collagen levels was dependent on GDH-driven glutamate generation, a reaction stemming from α-ketoglutarate. This notion is further supported by a recent study examining GLS1 inhibition in lung fibroblasts, which showed no reduction in oxygen consumption (indicative of electron transport chain [ETC] activity and, by proxy, of TCA cycle activity) following CB-839 treatment in pyruvate-replete conditions (31). Furthermore, we have previously published on the dispensable nature of the ETC for lung fibroblasts to mount a full TGF-β_1_–induced collagen response (34). Taken together, the evidence might now be mounting that biosynthetic processes might be more limiting than bioenergetic processes (such as NADH generation through the TCA cycle) for TGF-β_1_–enhanced collagen production.

Our results highlight alanine as a critical amino acid supporting TGF-β_1_–induced collagen synthesis. In the absence of extracellular alanine, siRNA-mediated silencing or pharmacological inhibition of GPT2 completely prevented pHLFs from mounting a full collagen response following TGF-β_1_ exposure. Of note, GPT2 knockdown did not affect baseline collagen levels, which suggests that targeting GPT2 therapeutically would preferentially limit collagen production in TGF-β_1_–activated fibroblasts. However, GPT2 expression was found to be very low in IPF myofibroblasts, and therefore a strategy to target extracellular alanine from the circulation could be modulated to limit fibrogenesis, as has been explored in the setting of cancer cell proliferation by intravenous administration of amino acid–degrading enzymes, such as asparaginase or cysteinase (35, 36).

Taken together, the findings presented herein shed important light on the complex interplay between metabolic networks and highlight a therapeutic strategy aimed at targeting both mitochondrial pyruvate metabolism and glutaminolysis to interfere with the pathological deposition of collagen in the setting of pulmonary fibrosis and potentially other fibrotic conditions.

## Methods

### Sex as a biological variable.

For this study we used a 1-dimensional cell culture model and an ex vivo human model to investigate disease mechanisms. We therefore did not consider sex as a biological variable.

### Cytokines, metabolites, and compounds.

The cytokine used was TGF-β_1_ (R&D Systems, Bio-Techne). Pharmacological inhibitors used were CB-839 (MedChemExpress), EGCG (MilliporeSigma), l-cycloserine (MilliporeSigma), BPTES (MilliporeSigma), and Compound 968 (MilliporeSigma). For metabolites, dimethyl 2-oxoglutarate, l-glutamic acid dimethyl ester hydrochloride, l-alanine, l-proline, sodium pyruvate, sodium lactate, and D-(+)-glucose were purchased from MilliporeSigma and l-glutamine from Thermo Fisher Scientific.

### pHLF isolation and culture.

pHLFs were derived from explant culture of lung tissue obtained with consent and institutional research ethics committee approval. The donor was described as being a White 51-year-old man who was a nonsmoker at the time of tissue acquisition. Cells were grown in standard DMEM free from glucose and glutamine, which were added individually to meet physiological concentrations of 5 mM and 0.7 mM, respectively, or in standard DMEM with 25 mM glucose, 1 mM pyruvate, and 2 mM glutamine. Media was supplemented with 10% fetal bovine serum (FBS) (MilliporeSigma) and 1% Penicillin (100 U/mL)/Streptomycin (100 μg/mL) (Thermo Fisher Scientific). All cultures were routinely screened for mycoplasma.

### Transcriptomic analysis by RNA-Seq.

Fibroblasts were grown to confluence in 6-well plates, and total RNA was extracted using RNeasy Kit (QIAGEN). Poly(A)-tailed RNA enrichment and library construction were performed using a stranded mRNA-Seq kit with mRNA capture beads (both KAPA Biosystems). High-throughput sequencing was performed at UCL Genomics using the NextSeq sequencing platform (Illumina), and the sequencing data were uploaded to the Galaxy web platform for analysis (37). Sequence reads were quality tested using FASTQC (version 0.72) and aligned to the hg38 human genome using HISAT2 with default parameters (version 2.1.0). FeatureCounts was used to quantify counts over reference genes (version 1.6.4), and differential gene expression was carried out using DESeq2 (version 2.11.40.6). DEGs were defined as having an adjusted *P* ≤ 0.05 and a log_2_ fold-change ≤ –0.58 or ≥ 0.58 when comparing 2 experimental conditions. Pathway enrichment analysis was carried out on normalized counts using clusterProfiler (version 3.17) and GSEA (version 4.1.0) with 1,000 permutations, a gene set size filter of 15–500, and the default metric for ranking genes.

### Quantification of amino acids using HPLC.

Free amino acids were quantified using HPLC. Fibroblast monolayers were washed once with ice-cold PBS before addition of ice-cold RIPA buffer (Thermo Fisher Scientific) supplemented with protease inhibitors (cOmpleteMini, Roche). Lysates were then transferred to 1.5 mL Eppendorf tubes and vortexed vigorously for 30 seconds prior to centrifugation at 21,000*g* for 10 minutes at 2°C. Supernatants were then transferred to 3 kDa protein filter columns (MilliporeSigma) and centrifuged at 12,000*g* for 45 minutes. The flow-through was then dried in a speed-vac and derivatized with 7-chloro-4-nitrobenzo-2-oxa-1,3-diazole (Acros Organics, Thermo Fisher Scientific) prior to reverse-phase HPLC (Agilent 1100 series, Agilent Technologies) for hydroxyproline isolation using acetonitrile as the organic solvent in a LiChrospher, 100 RP-18 column.

### Quantification of hydroxyproline from cellular supernatants using HPLC.

Fibroblast pro-collagen secretion was assessed by HPLC quantification of hydroxyproline in cell supernatants. Cells were cultured in starving (0.4% FBS) media supplemented with 25 μg/mL of l-ascorbic acid 2-phosphate sesquimagnesium salt hydrate (Merck Millipore) to ensure appropriate ascorbic acid availability for collagen hydroxylation reactions. Proteins were precipitated by adding ethanol to a concentration of 66% (v/v), vortexed, and left overnight at 4°C. The solutions were then filtered using 0.45 μm filters (MilliporeSigma), which were then incubated with 6 M hydrochloric acid at 110°C for 16 hours. Samples were then dried at 100°C and derivatized with 7-chloro-4-nitrobenzo-2-oxa-1,3-diazole prior to reverse-phase HPLC for hydroxyproline isolation using acetonitrile as the organic solvent in a 100 RP-18 column.

### High-content imaging for collagen type I deposition analysis.

Collagen type I deposition was measured in 96-well plates using high-content immunofluorescence-based imaging in molecular crowding conditions as previously described (16). Briefly, pHLFs were grown in a 96-well plate in 10% FBS media until confluence before replacement with 0.4% FBS media for 24 hours. Media were then replaced once more with 0.4% FCS containing ascorbic acid (16.6 μg/mL) and Ficoll 70 and Ficoll 400. Cells were then treated with compounds for 2 hours prior to TGF-β_1_ stimulation (1 ng/mL, R&D Systems, Bio-Techne). After 48 hours, the media were removed, and monolayers were fixed with methanol prior to antibody addition targeting human collagen I (MilliporeSigma) followed by Alexa Fluor 488 secondary antibody (Life Technologies, Thermo Fisher Scientific A11001) and nuclear DAPI counterstain. Fluorescence was quantified using an ImageXpress Micro XLS high-content imaging system at 20× original magnification (Molecular Devices) with 4 fields of view per well and collagen signal normalized to cell count.

### Immunoblotting.

pHLFs were lysed using ice-cold PhosphoSafe buffer (Merck Millipore) supplemented with protease inhibitors (cOmpleteMini). Protein concentration was determined using a Pierce BCA protein assay kit (Thermo Fisher Scientific), and 10 μg protein was loaded into 15-well NuPage 4%–12% Bis-Tris gels (Thermo Fisher Scientific) for SDS-PAGE separation. Gels were then transferred to nitrocellulose, and protein levels were assessed by Western blotting with the following antibodies: GLS1 (ProteinTech 12855-1-AP), GPT2 (Atlas Antibodies HPA051514), GOT2 (Thermo Fisher Scientific PA5-27572), GOT1 (CST 34423), P5CS (Abcam ab223713), SHMT2 (Cell Signaling Technology [CST] 12762), ASNS (ProteinTech 14681-1-AP), GDH (ProteinTech 14299-1-AP), PSAT1 (Thermo Fisher Scientific PA5-22124), and α-tubulin (CST 9099). Dilutions were 1:1,000, except for α-tubulin, which was 1:5,000. All densitometries are presented relative to α-tubulin unless stated otherwise.

### siRNA-mediated protein expression knockdown.

pHLFs were seeded in a 12-well plate in 10% FBS media for 24 hours before transfection with 25 nM siRNAs (Dharmacon, SMARTpool) targeting *GPT2*, *GOT1*, *GOT2*, *PSAT1*, *SHMT2*, *ASNS*, *GDH*, and *ALDH18A1* using RNAiMax Lipofectamine (Invitrogen, Thermo Fisher Scientific) following the manufacturer’s recommendations. The following day the media were replaced with 0.4% FBS media and left for a further 24 hours before TGF-β_1_ treatment.

### Metabolomic analysis and labeling using LC-MS.

pHLFs were plated in 6-well plates and grown to confluence. For labeling studies, ^13^C_6_-glucose or ^13^C_3_-pyruvate was added for either the full 48 hours or in the last 8 hours accompanied by a media change (Cambridge Isotope Laboratories). Cells were then washed with ice-cold PBS 3 times and metabolites extracted using an extraction solvent consisting of 50% methanol, 30% acetonitrile, and 20% ultrapure water added with 100 ng/mL of HEPES. Cells were scraped and transferred to a shaker for 15 minutes at 4°C before an incubation at –20°C for 1 hour. Samples were then spun at maximum speed, 16,000*g*, for 10 minutes at 4°C, and supernatants were transferred to new tubes (process repeated twice). Supernatants were then transferred to autosampler glass vials and stored at –80°C until analysis. Samples were then loaded onto the LC-MS machine in a randomized order to avoid machine drift bias. LC-MS analysis was performed using a Q Exactive Hybrid Quadrupole-Orbitrap mass spectrometer coupled to a Dionex U3000 UHPLC system (Thermo Fisher Scientific). A Sequant ZIC-pHILIC column (150 mm × 2.1 mm) was used with a guard column (20 mm × 2.1 mm) (Merck Millipore). The temperature was maintained at 45°C, and mobile phase consisted of 20 mM ammonium carbonate and 0.1% acetonitrile. Flow rate was 200 μL/min with previously described gradient (17). The mass spectrometer was used in full MS and polarity-switching mode. Spectra were analyzed using Xcalibur Qual Browser and Xcalibur Quan Browser software (Thermo Fisher Scientific). The mzCloud advanced mass spectral database was used for the untargeted analysis.

### mRNA quantification using real-time quantitative PCR.

RNA was extracted using RNeasy Mini kit (QIAGEN) following the manufacturer’s instructions. Real-time PCR was performed using a Mastercycler Realplex ep gradient S (Eppendorf). Amplification was performed for 40 cycles with a melting temperature of 95°C for 15 seconds and an annealing temperature of 60°C for 1 minute. Relative quantification was derived using 2-ΔCt (ΔCt calculated from the mean of 2 normalizing genes, *ATP5B* and *B2M*; PrimerDesign Ltd, primer sequence proprietary). The following primers were used: GPT2 forward 5′-TCCTCACGCTGGAGTCCATGA-3′ and reverse 5′-ATGTTGGCTCGGATGACCTCTG-3′; GDH (GLUD1) forward 5′-CTCCAAACCCTGGTGTCATT-3′ and reverse 5′-CACACGCCTGTGTTACTGGT-3′.

### Pyruvate quantification using enzymatic assay.

Intracellular pyruvate levels following lactate supplementation were quantified using an enzyme-based fluorometric assay (ab65342 Abcam), and the protocol was followed according to the manufacturer’s instructions.

### PCLS.

Freshly obtained lung tissue was immersed in alginate (3% w/v), and CaCl_2_ (3% w/v) was added immediately to create an artificial pleura as previously described (18). Lung tissue was inflated with a 2% (w/v) agarose solution using a 22G syringe. The inflated tissue fragment was then placed in a Peel-A-Way embedding mold (E6032, Merck) and filled with 4% (w/v) agarose solution until solidified. The tissue was then placed in HBSS solution (Gibco, Thermo Fisher Scientific) supplemented with 1% (v/v) of penicillin/streptomycin and cut with a Leica VT1200S microtome set at 1.5 mm/s, 3 mm, to yield 400 μm slices. Slices were then individually placed in a well of a 6-well plate with 3 mL of 0.4% FBS DMEM^hi^. Slices used for the determination of viability were cut and placed into a black-walled, 96-well plate, and CellTiter-Blue reagent (Promega) was added following the manufacturer’s instructions. Following 24 hours, media were changed to 0.4% FBS DMEM^lo^ with pyruvate (1 mM) added, and 10 ng/mL of TGF-β_1_ was used to initiate the fibrotic program.

### Immunofluorescence of IPF lung tissue.

Immunostaining for GLS1, GDH, and α-SMA was performed on 10 μm, formalin-fixed, paraffin-embedded serial sections of IPF lung biopsy material. Sections were dewaxed and permeabilized with 0.3% Triton X-100 in PBS for 10 minutes before antigen retrieval using 10 mM citrate buffer pH 6 and heated at full power in a microwave. Slides were then blocked with 5% normal goat serum in 0.1% Triton X-100 in PBS for 1 hour at room temperature. Primary antibodies (α-SMA, clone 1A4 Agilent Dako, Thermo Fisher Scientific; GLS1, ab260047 Abcam; GHD, ab168352 Abcam) were then added at a 1:200 dilution in blocking buffer and left overnight at 4°C. Following PBS washes, secondary antibodies at 1:1,000 in 0.1% Triton X-100 PBS were added for 3 hours at room temperature (anti-rabbit Alexa Fluor 488, Invitrogen A-11008; and anti-mouse Alexa Fluor 555, A-31570, Invitrogen, Thermo Fisher Scientific). Following PBS washes, DAPI in PBS 1:10,000 was added for 20 minutes before PBS washing and mounting using Fluoromount-G (Life Technologies, Thermo Fisher Scientific). Fluorescence images were then captured using a DMi8 S inverted microscope (Leica Microsystems).

### Statistics.

All data are expressed as the means ± SD and generated using GraphPad Prism version 8.0, and all experiments are repeated at least 3 times. Statistical differences between 2 groups were determined using 1-way or 2-way ANOVA with Tukey’s post hoc test. Four-parameter and nonlinear regression analysis was used to produce IC_50_ values from compound concentration curves. The α level was 0.05 for all tests.

### Study approval.

pHLFs were derived through explant culture and tissue obtained with informed and written consent and with institutional research ethics committee approval from the UCL Research Ethics Committee (12/EM/0058). IPF lung tissue were obtained from patients undergoing lung transplantation or surgical lung biopsy following informed written consent and with research ethics committee approval (10/H0504/9—National Institute of Health Biomedical Research Unit Advanced Disease Biobank, Royal Brompton Hospital, 10/H0720/12—London—Hampstead Research Ethics Committee). Tissue for PCLS experiments was obtained from UCL Hospitals in compliance with the UK Human Tissue Act 2004, and ethical approval was obtained through the National Research Ethics Committee (18/SC/0514)- South Central, Hampshire B Research Ethics Committee administered through University College London Hospitals NHS Foundation Trust.

### Data availability.

The RNA-Seq dataset has been deposited and is available at NCBI GEO GSE244098. All other data needed to evaluate the conclusions in the paper are present in the paper, Supporting Data Values file, or publicly available datasets in GEO as specified in Results.

## Author contributions

GC conceptualized the approach, performed experiments, interpreted the data, generated the figures, and collaboratively drafted the manuscript. RCC conceived the study, secured the funding, and supervised the work. JAAMW and GC performed the bioinformatics analysis of RNA-Seq datasets. MP and DG performed experiments and provided RNA-Seq datasets. BS helped edit the manuscript. VM performed the LC-MS analysis for the metabolomics study under the supervision of KB, who also interpreted data. VP and MT provided expert input into the study. All authors reviewed and approved the final submitted manuscript.

## Supplementary Material

Supplemental data

Supplemental data set 1

Unedited blot and gel images

Supporting data values

## Version 1 09/24/2024

Electronic publication
