# Supplementary material for: Pyruvate metabolism dictates fibroblast sensitivity to GLS1 inhibition during fibrogenesis
Source: JCI Insight. 2024 Aug 13;9(18):e178453. doi: 10.1172/jci.insight.178453 (PMC11457851; doi:10.1172/jci.insight.178453)
Supplement: Supplemental data [file jciinsight-9-178453-s119.pdf]

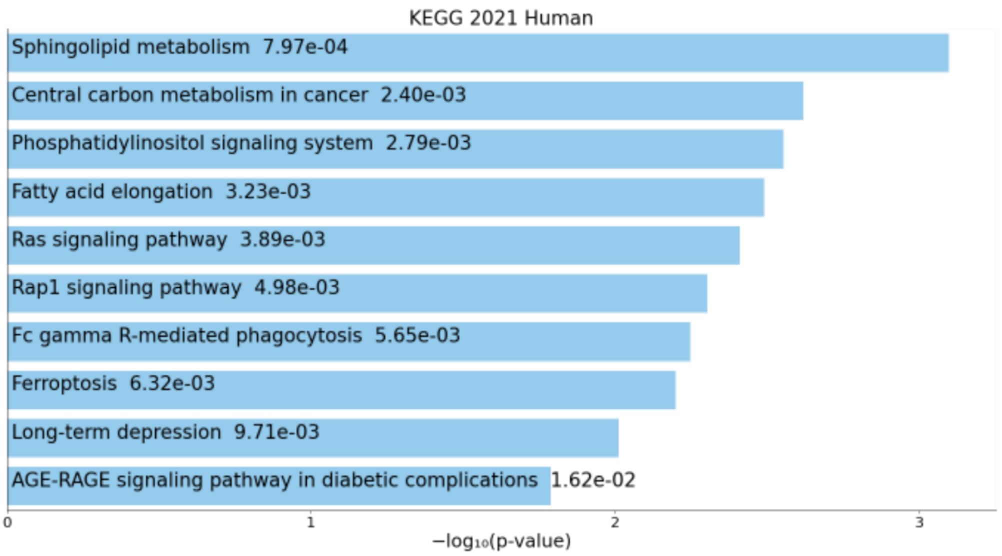


**Supplementary Figure 1. KEGG pathway analysis of DEGs unique to pHLFs cultured in DMEM$^{High}$.**

DEGs derived from RNA-Seq analysis of pHLFs 24 hours following TGF-$\beta_1$ (1 ng/ml) stimulation in DMEM$^{High}$ and DEGs found in DMEM$^{Low}$ were removed, leaving DEGs unique to DMEM$^{High}$.

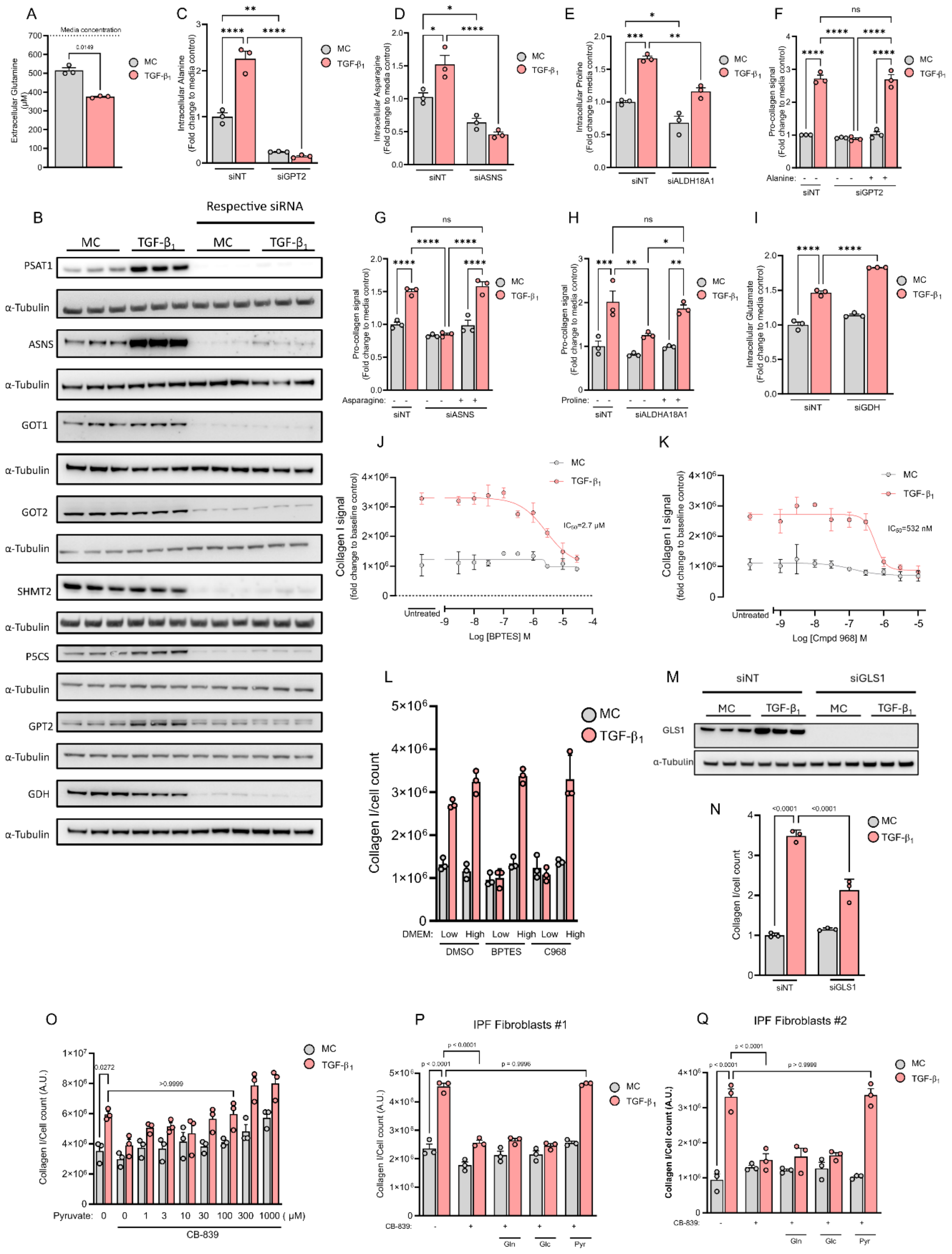
A
Media concentration
0.0149
Extracellular Glutamine (µM)
MC
TGF-β1
C
Intracellular Alanine (Fold change to media control)
siNT
siGPT2
D
Intracellular Asparagine (Fold change to media control)
siNT
siASNS
E
Intracellular Proline (Fold change to media control)
siNT
siALDH18A1
F
ns
Pro-collagen signal (Fold change to media control)
Alanine:
siNT
siGPT2
B
Respective siRNA
MC
TGF-β1
PSAT1
α-Tubulin
ASNS
GOT1
GOT2
SHMT2
P5CS
GPT2
GDH
G
Pro-collagen signal (Fold change to media control)
Asparagine:
siNT
siASNS
H
Pro-collagen signal (Fold change to media control)
Proline:
siNT
siALDHA18A1
I
Intracellular Glutamate (Fold change to media control)
siNT
siGDH
J
Collagen I signal (fold change to baseline control)
IC50=2.7 µM
Untreated
Log [BPTES] M
K
Collagen I signal (fold change to baseline control)
IC50=532 nM
Untreated
Log [Cmpd 968] M
L
Collagen I/cell count
DMEM: Low High Low High Low High
DMSO
BPTES
C968
M
siNT
siGLS1
GLS1
α-Tubulin
N
<0.0001
Collagen I/cell count
siNT
siGLS1
O
Collagen I/Cell count (A.U.)
0.0272
>0.9999
Pyruvate: 0 0 1 3 10 30 100 300 1000 ( µM)
CB-839
P
IPF Fibroblasts #1
p < 0.0001
p = 0.9996
Collagen I/Cell count (A.U.)
CB-839:
Gln
Glc
Pyr
Q
IPF Fibroblasts #2
p < 0.0001
p > 0.9999
Collagen I/Cell count (A.U.)
CB-839:
Gln
Glc
Pyr

**Supplementary Figure 2. Metabolite rescues of TGF-$\beta_1$-induced collagen under metabolic enzyme inhibition or protein expression knockdown.** **(A)** Extracellular glutamine levels 48 hours following TGF-$\beta_1$ (1 ng/ml) in DMEM$^{Low}$. **(B)** pHLFs were transfected with non-targeting (NT) siRNA or targeting siRNA and protein expression measured by immunoblot 24 hours following TGF-$\beta_1$ (1 ng/ml). **(C-E)** pHLFs were stimulated with TGF-$\beta_1$(1 ng/ml) for 48 h following transfection with non-targeting siRNA for control or siRNA targeting GPT2, ASNS or ALDH18A1 and intracellular levels of alanine, asparagine or proline (respectively) quantified using HPLC. (**F-H)** Media supplemented with respective metabolite (500 µM) and hydroxyproline quantified using HPLC (*n*=3). **(I)** pHLFs were treated as in **C-E** and intracellular glutamate quantified with HPLC. **(J** and **K)** pHLFs were grown in DMEM$^{Low}$ and pre-incubated with increasing concentrations of BPTES or Compound 968 for 1 h before TGF-$\beta_1$ (1 ng/ml) for 48 h and collagen I deposition assessed by macromolecular crowding (MMC) assay. **(L)** pHLFs were grown in DMEM$^{Low}$ or DMEM$^{High}$ and treated with BPTES (10 µM) or Compound 968 (1 µM) before being stimulated with TGF-$\beta_1$ (1 ng/ml) for 48 h and collagen I deposition assessed by MMC assay. **(M** and **N)** Immunoblot showing siGLS1 effect on GLS1 protein abundance as in **B** and **N** collagen quantified by macromolecular crowding assay 48 hours following TGF-$\beta_1$ (1 ng/ml) stimulation. **(O)** pHLFs were grown in DMEM$^{Low}$ and pre-incubated with increasing concentrations of pyruvate for 1 h before being stimulated with TGF-$\beta_1$ (1 ng/ml) and treated with CB-839 (1 µM) for 48 h and collagen I deposition assessed by MMC assay. **(P** and **Q)** Two IPF fibroblast cell lines were grown in DMEM$^{Low}$ supplemented with glutamine (1.3 mM), glucose (20 mM) or pyruvate (1 mM) before pre-incubation with 1 µM CB-839 for 1 h before TGF-$\beta_1$ (1 ng/ml) stimulation for 48 h and collagen I deposition assayed by MMC assay. Data are presented as mean ± SD and differences evaluated between groups with two-way ANOVA with Tukey multiple comparison testing. *=p<0.05, **=p<0.01, ***=p<0.001, ****=p<0.0001.

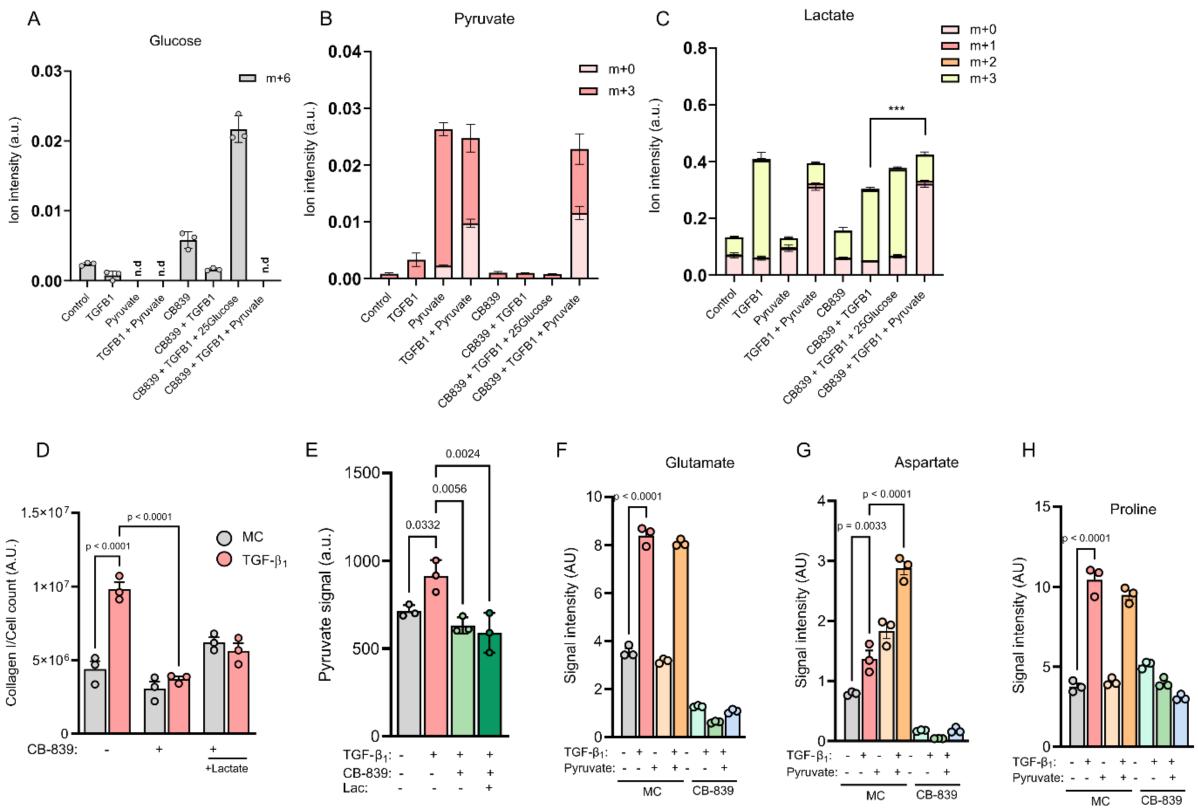


**Supplementary Figure 3. High glucose does not phenocopy exogenous pyruvate. (A** and **B)** and **(F** and **H)** Intracellular isotopologue levels and **F-H** total abundance of specified metabolite in pHLFs grown in DMEM$^{Low}$ supplemented with U-$^{13}$C-glucose (5 mM) or U-$^{13}$C-pyruvate (1 mM) and pre-incubated with media control (0.1% DMSO) or 1 µM CB-839 for 1 h before stimulation with TGF-β$_1$ (1 ng/ml) for 48 h and quantification achieved using LC-MS *(n*=3). **(D)** Collagen deposition quantified 48 hours after TGF-β$_1$ (1 ng/ml) stimulation from pHLFs growing in DMEM$^{Low}$ and 1 µM CB-839 with supplementation of lactate (10 mM). **e** Intracellular pyruvate levels following 48 hours of treatment as in **D**. Data are presented as mean ± SD and differences evaluated between groups with two-way ANOVA with Tukey multiple comparison testing.
