# Supplementary material for: Pyruvate metabolism dictates fibroblast sensitivity to GLS1 inhibition during fibrogenesis
Source: JCI Insight. 2024 Aug 13;9(18):e178453. doi: 10.1172/jci.insight.178453 (PMC11457851; doi:10.1172/jci.insight.178453)
Supplement: Unedited blot and gel images [file jciinsight-9-178453-s121.pdf]

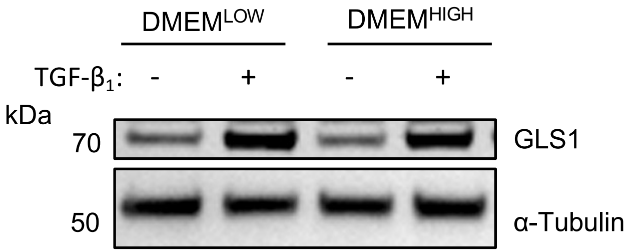

12855-1-AP

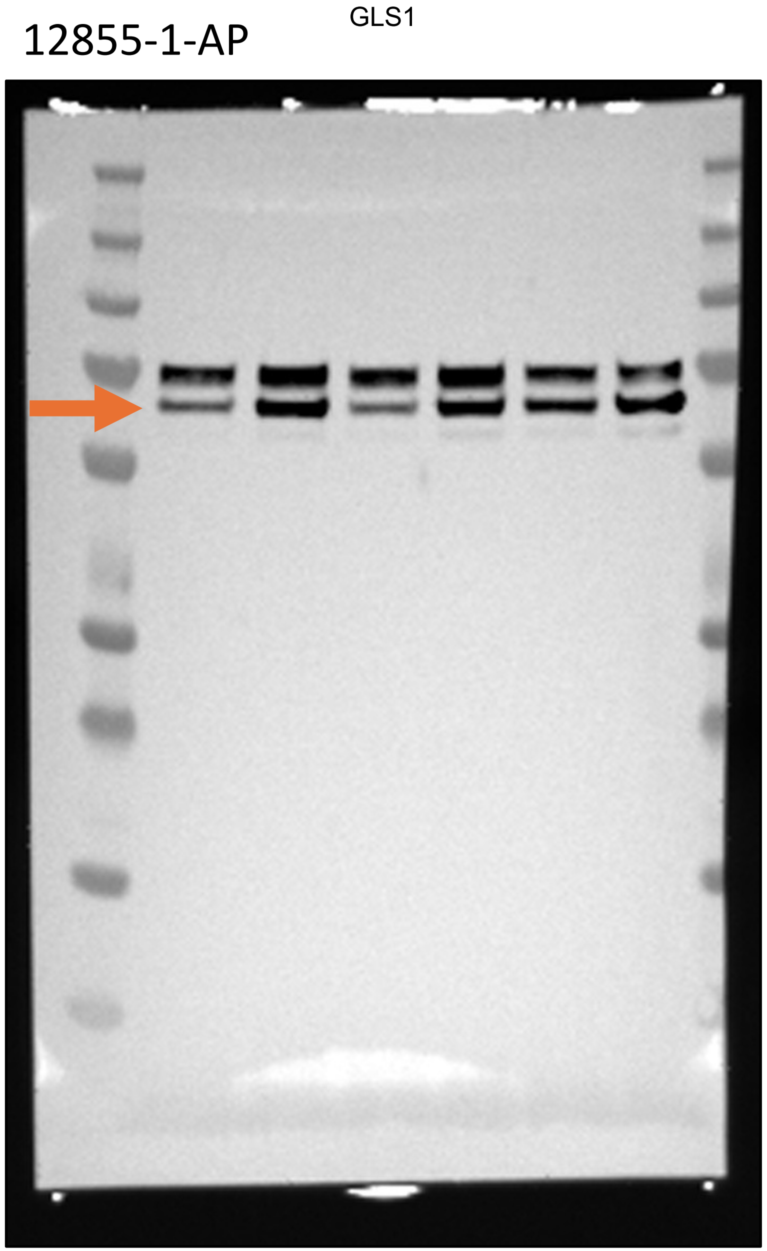

CST#9099

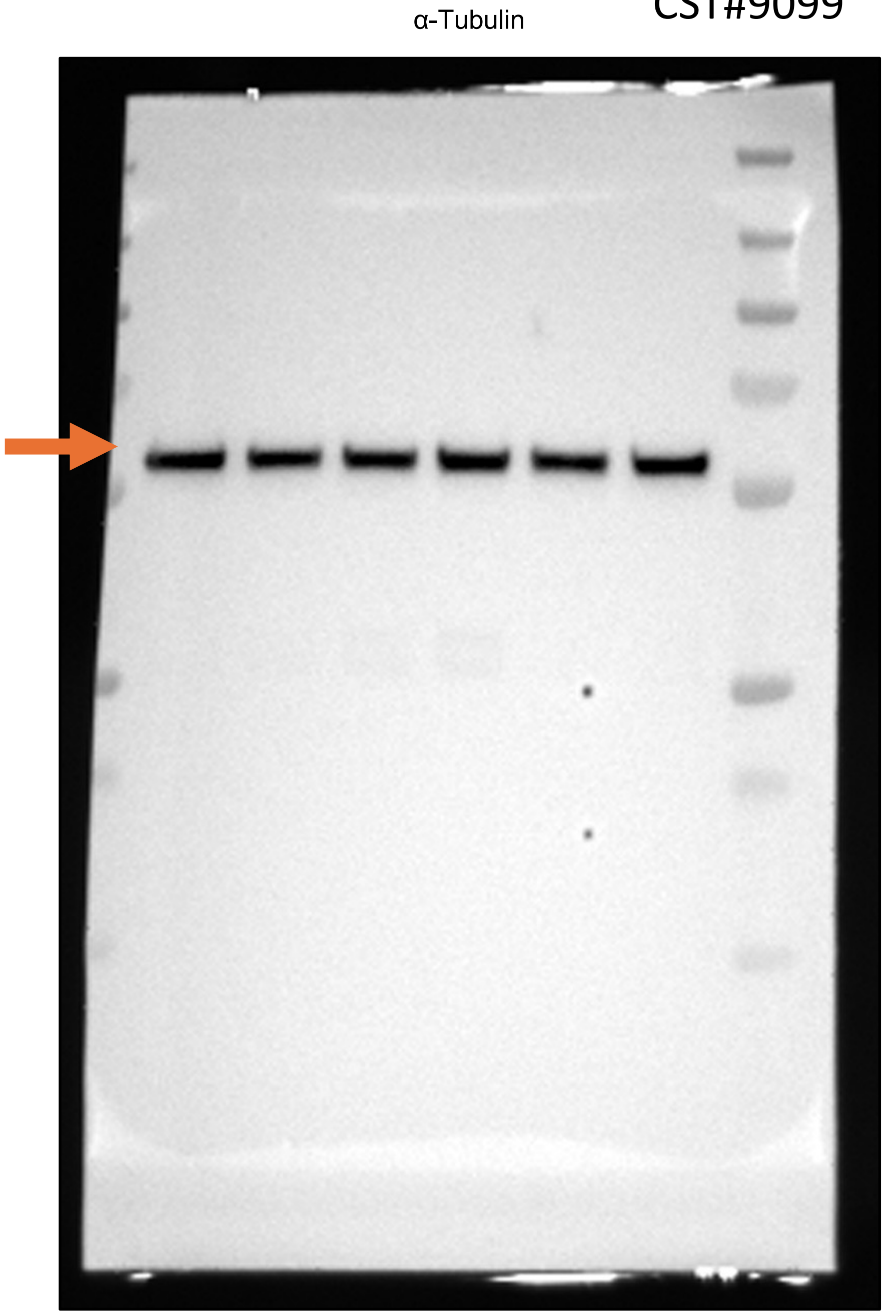

Full unedited gels for Fig 2g

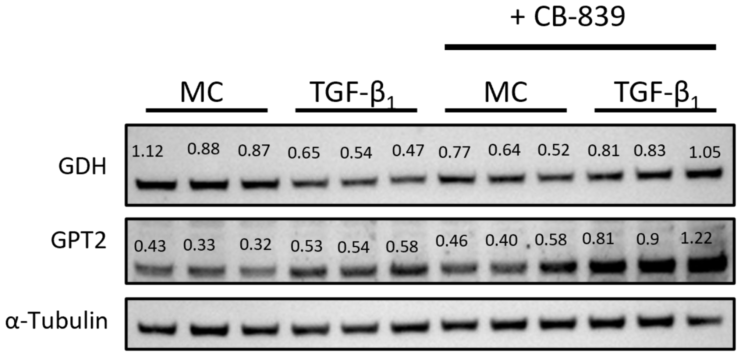

14299-1-AP GDH

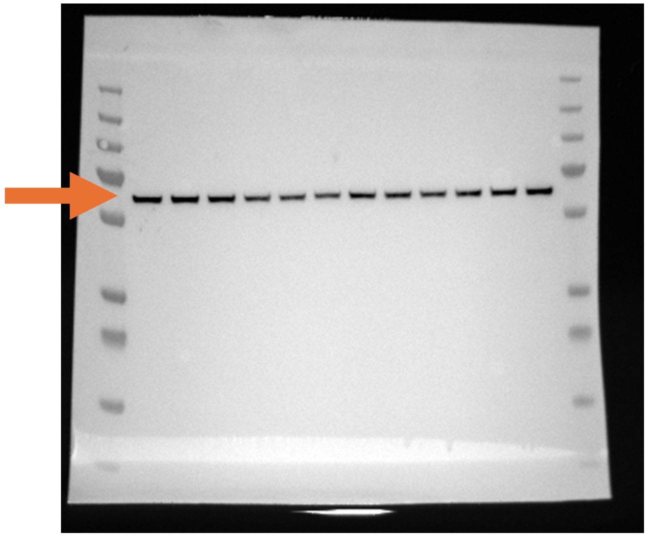

HPA051514 GPT2

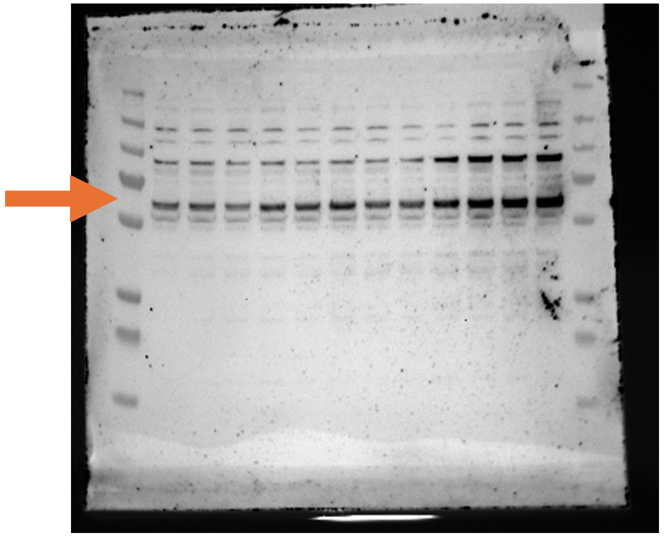

α-Tubulin CST#9099

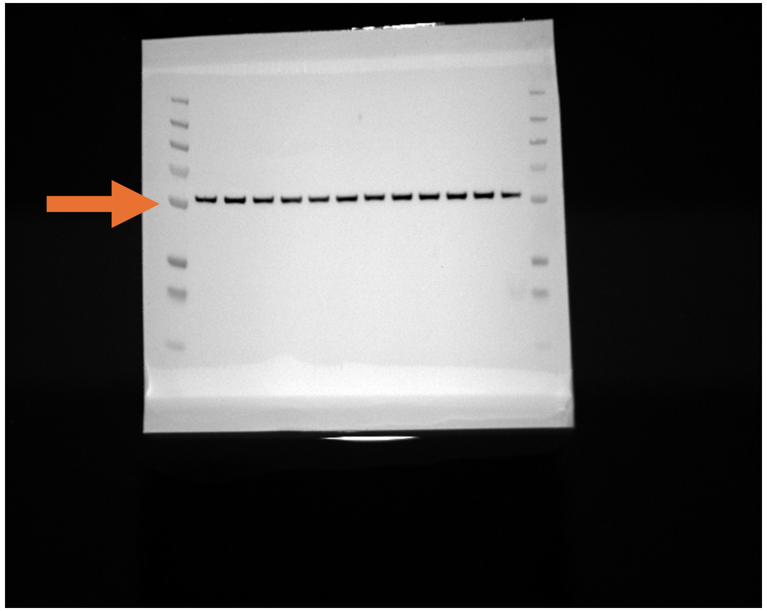

Full unedited gels for Fig 5g

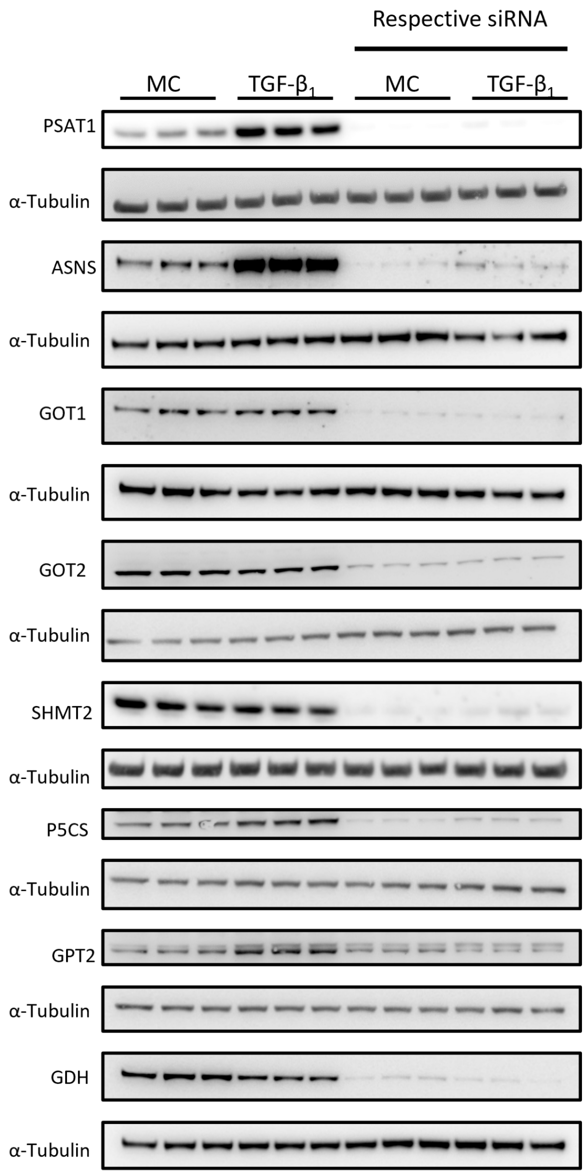

PSAT1

PA522124

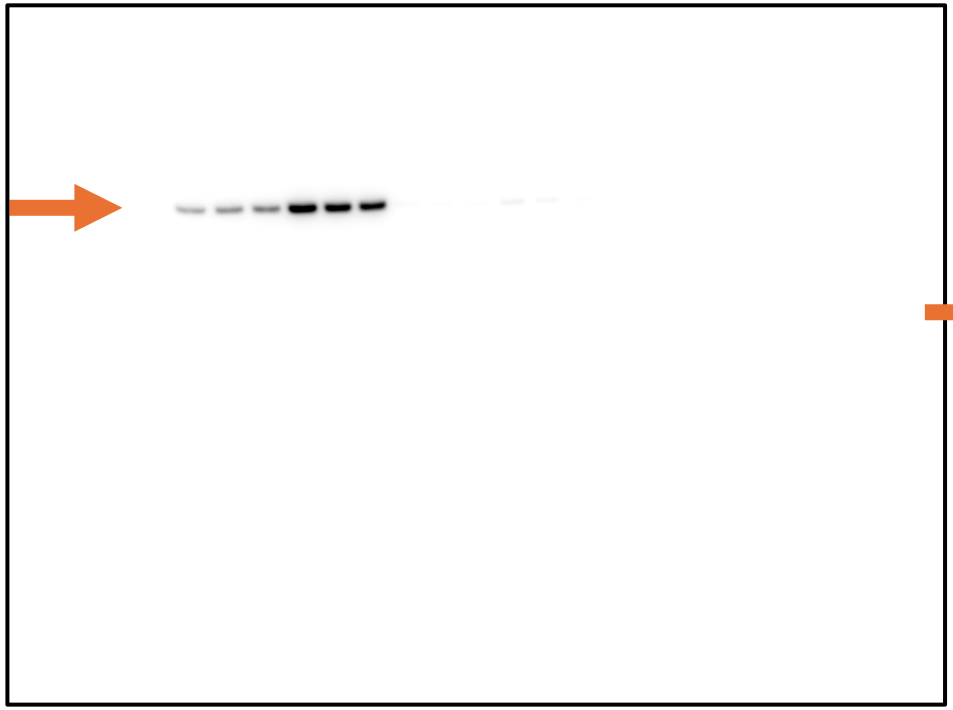

α-Tubulin

CST#9099

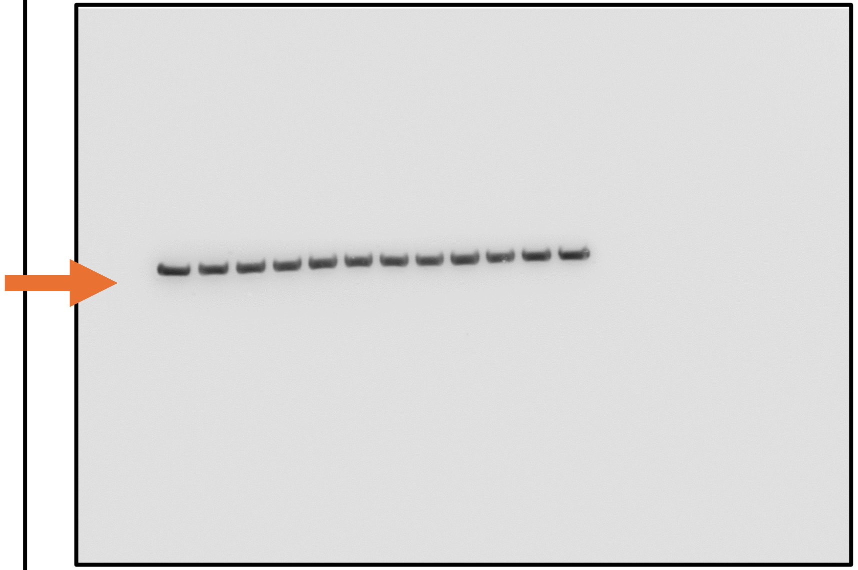

Full unedited gels for Fig s2b

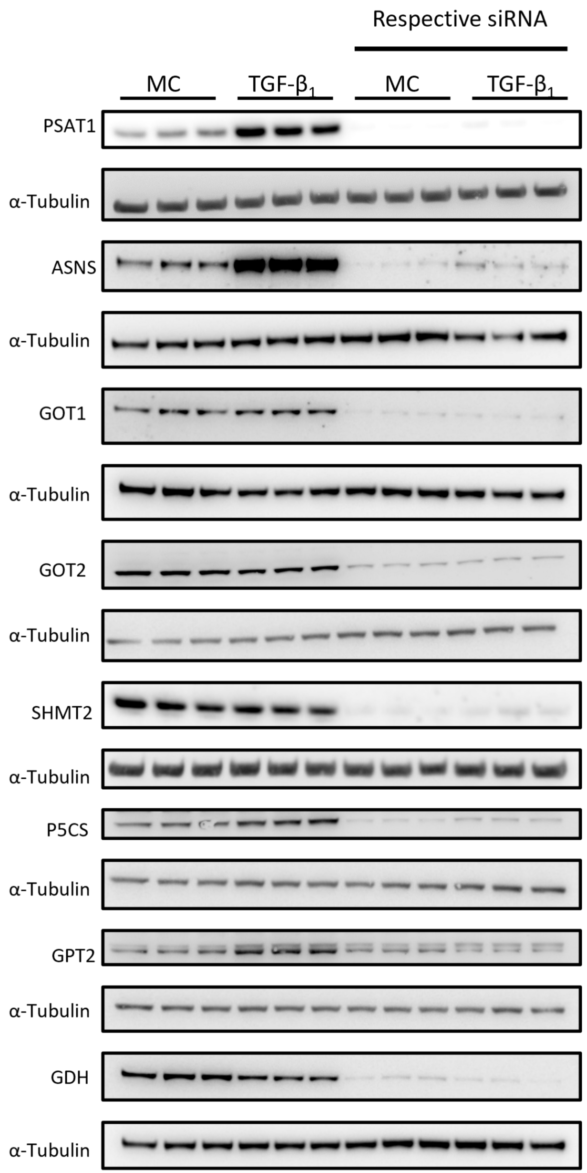

ASNS

14681-1-AP

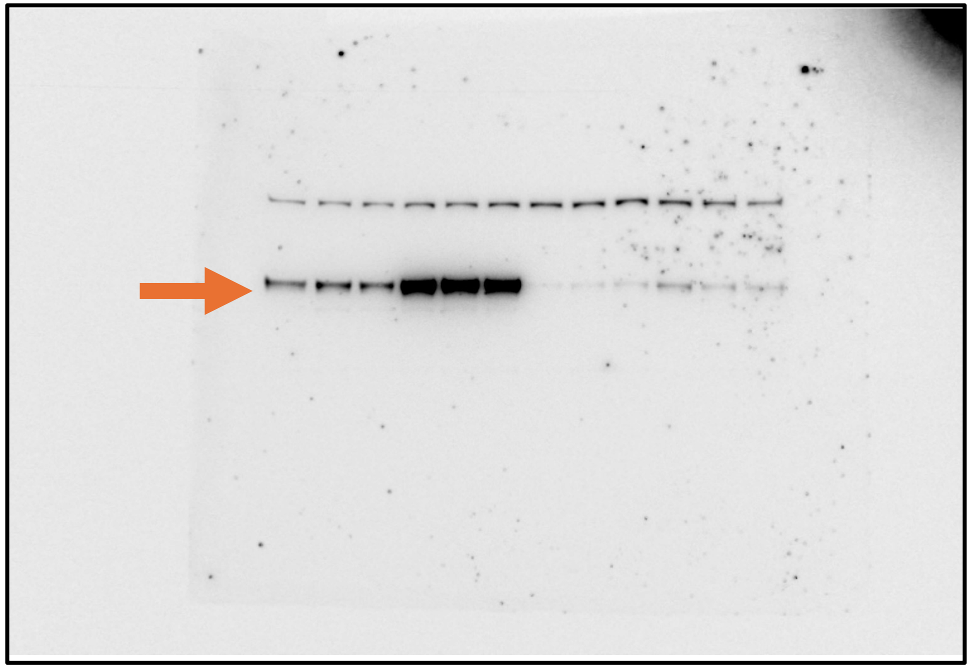

α-Tubulin

CST#9099

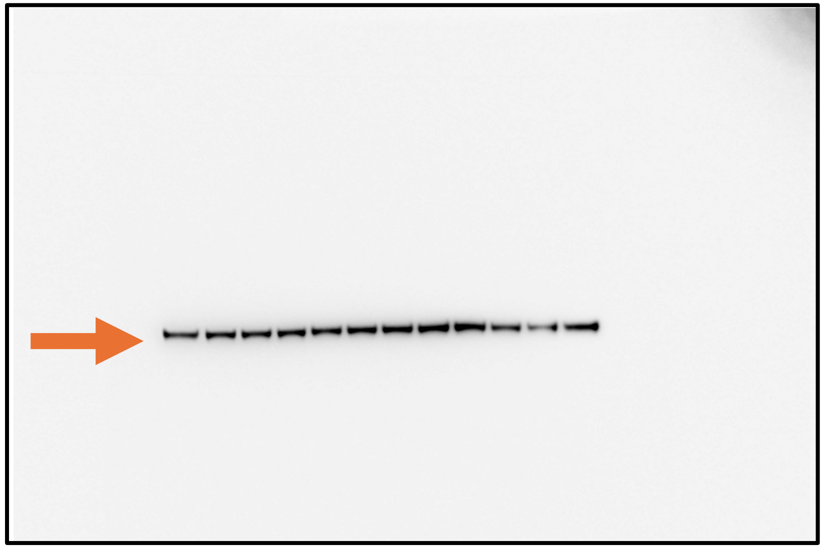

Full unedited gels for Fig s2b

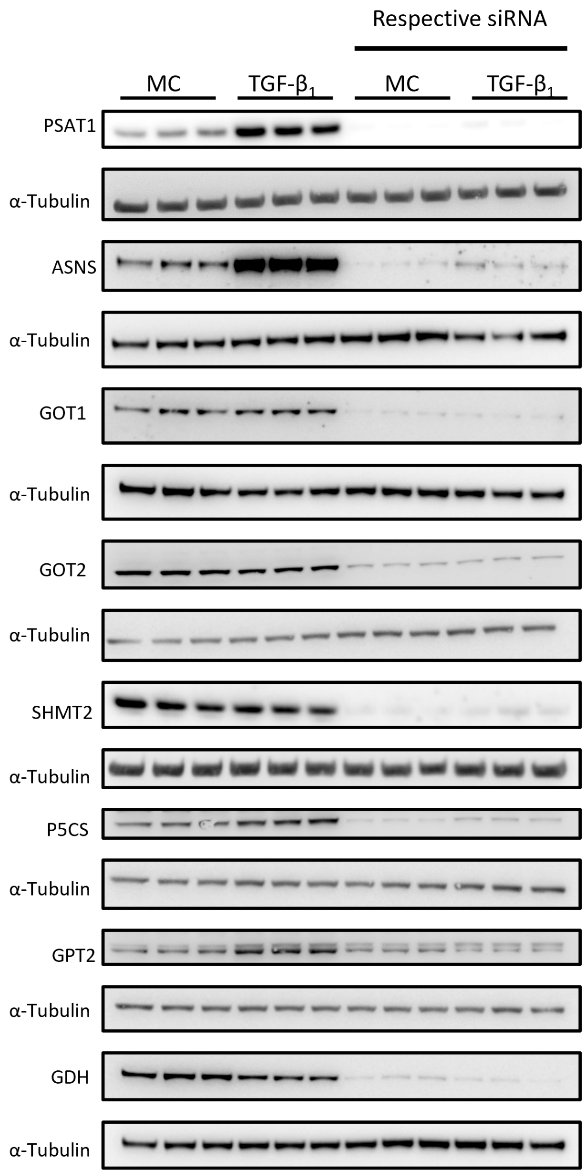

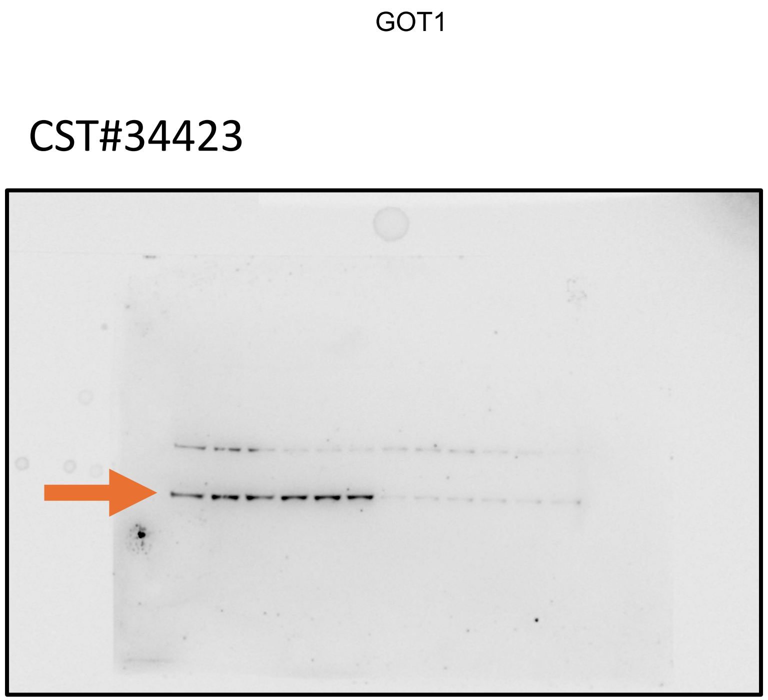

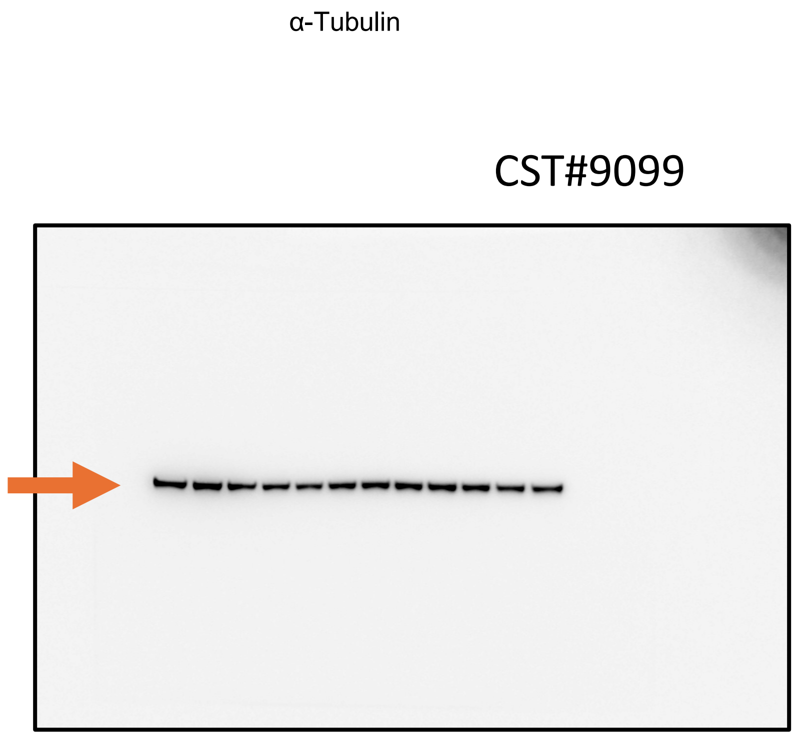

Full unedited gels for Fig s2b

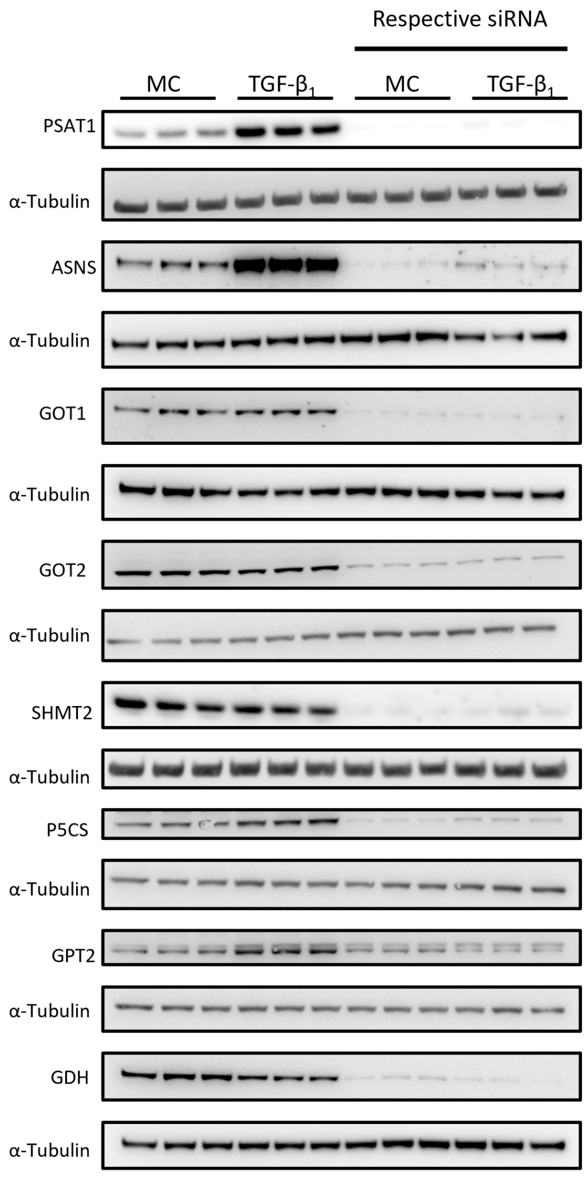

GOT2

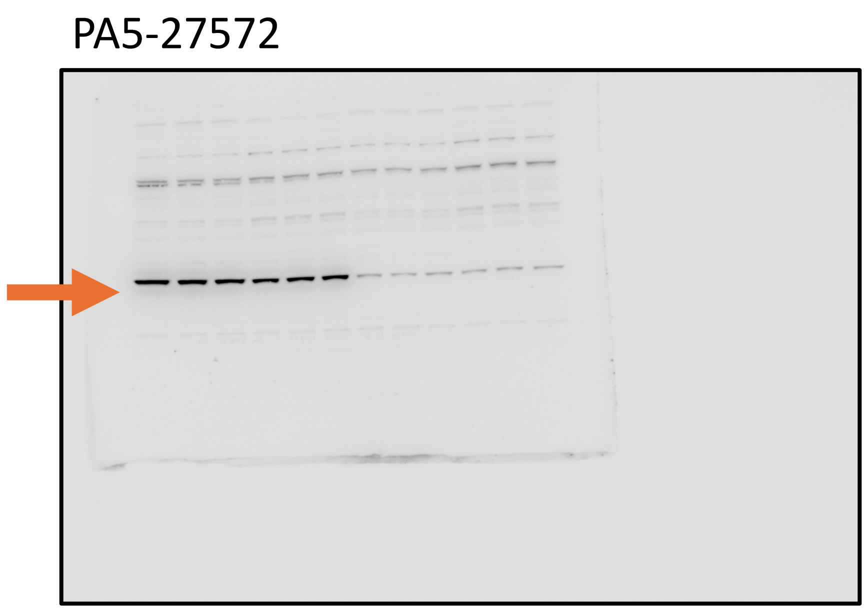

α-Tubulin

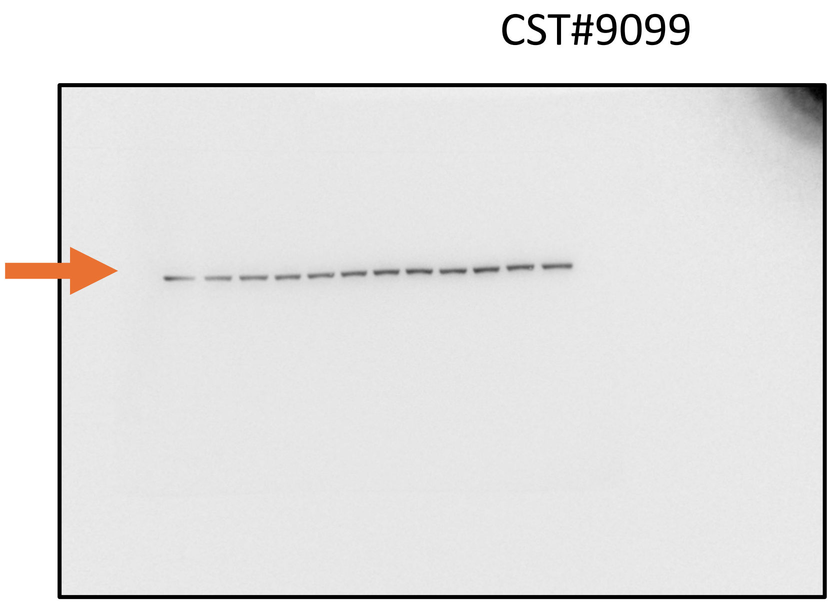

Full unedited gels for Fig s2b

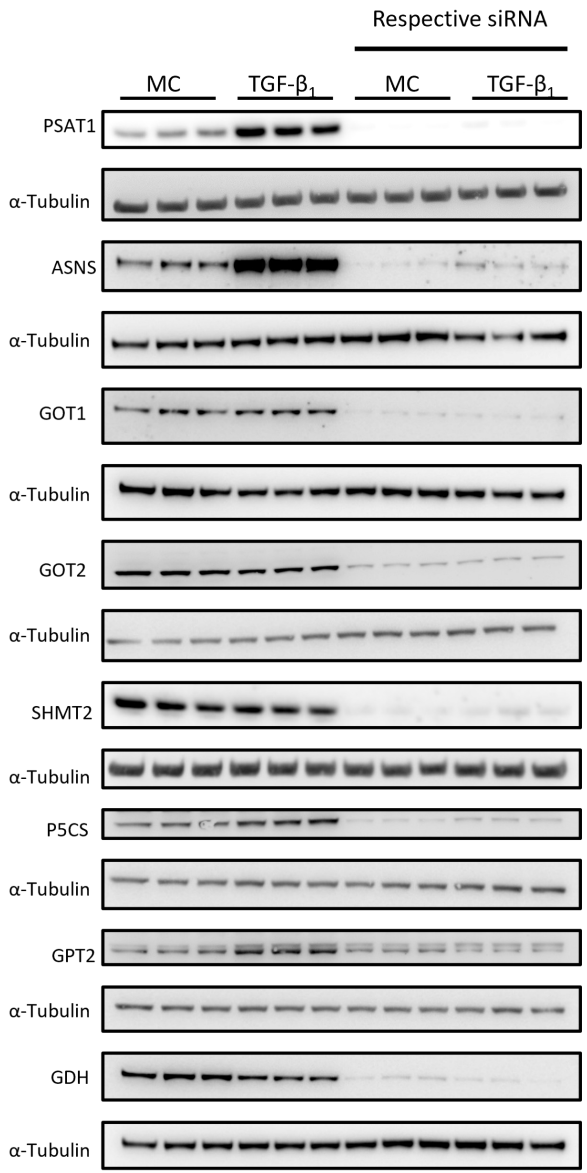

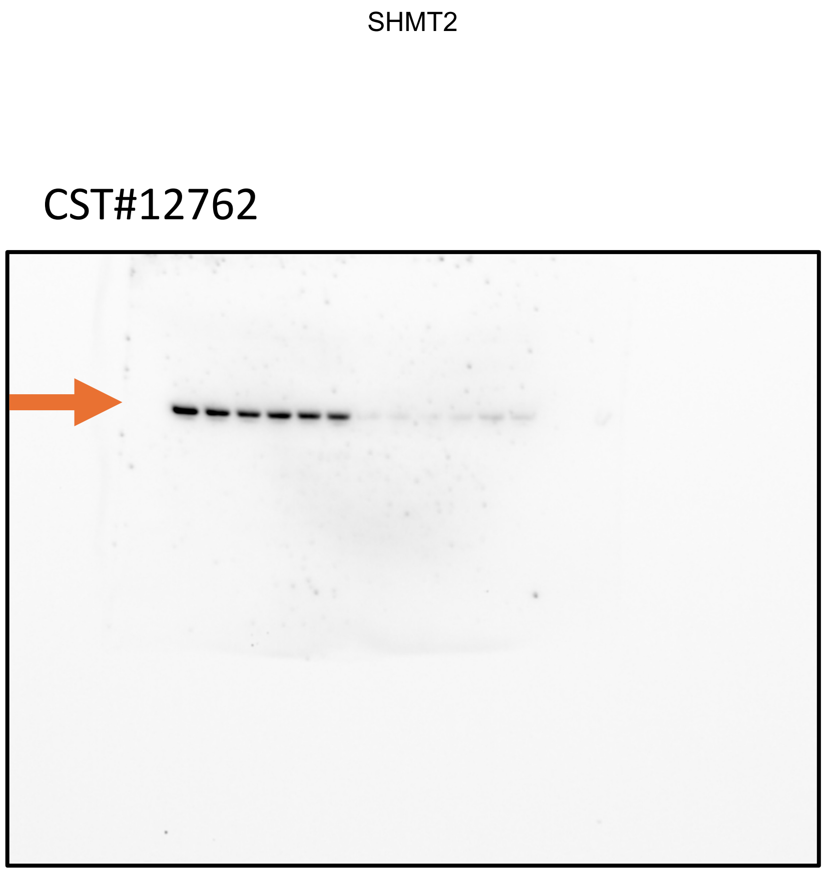

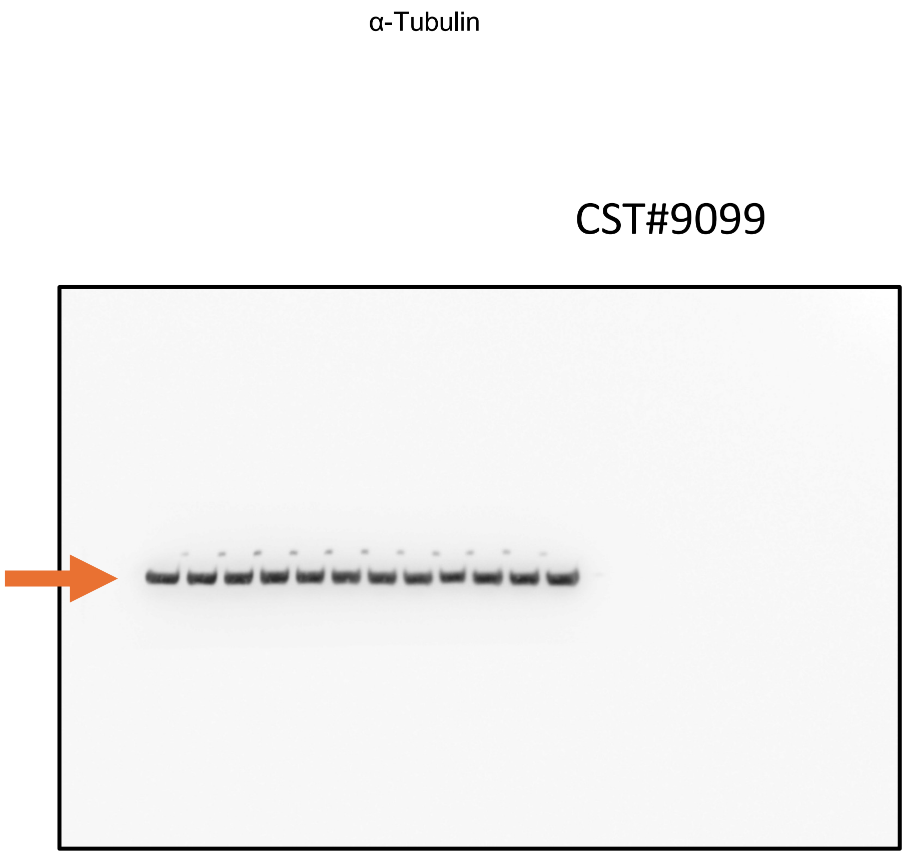

Full unedited gels for Fig s2b

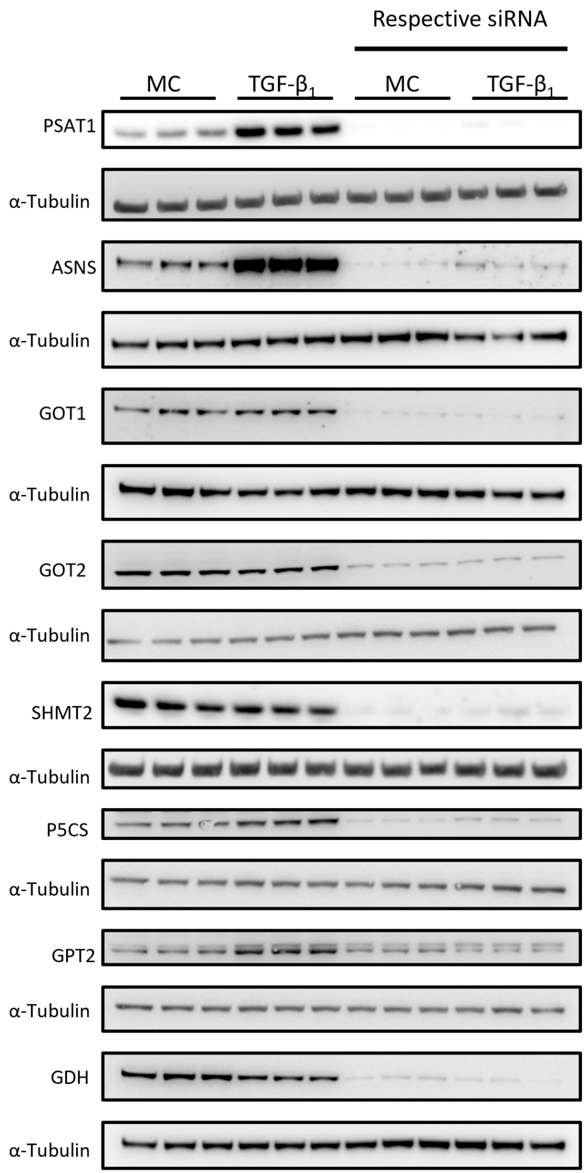

P5CS

ab223713

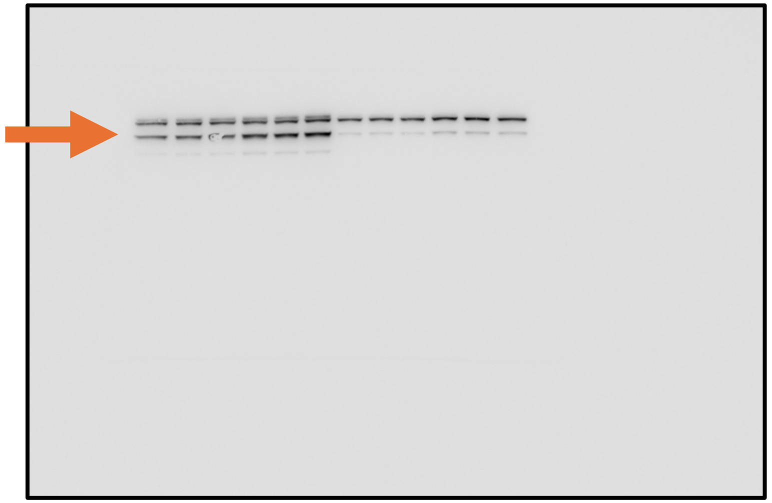

α-Tubulin

CST#9099

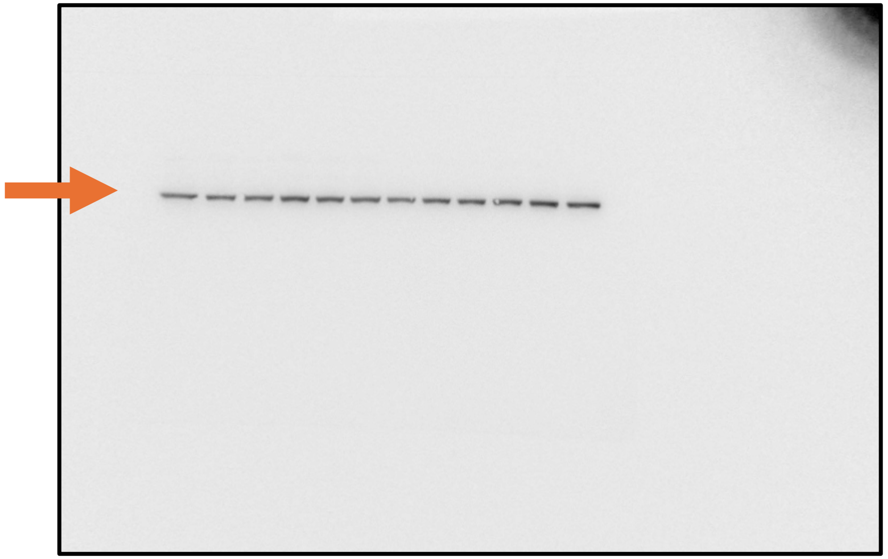

Full unedited gels for Fig s2b

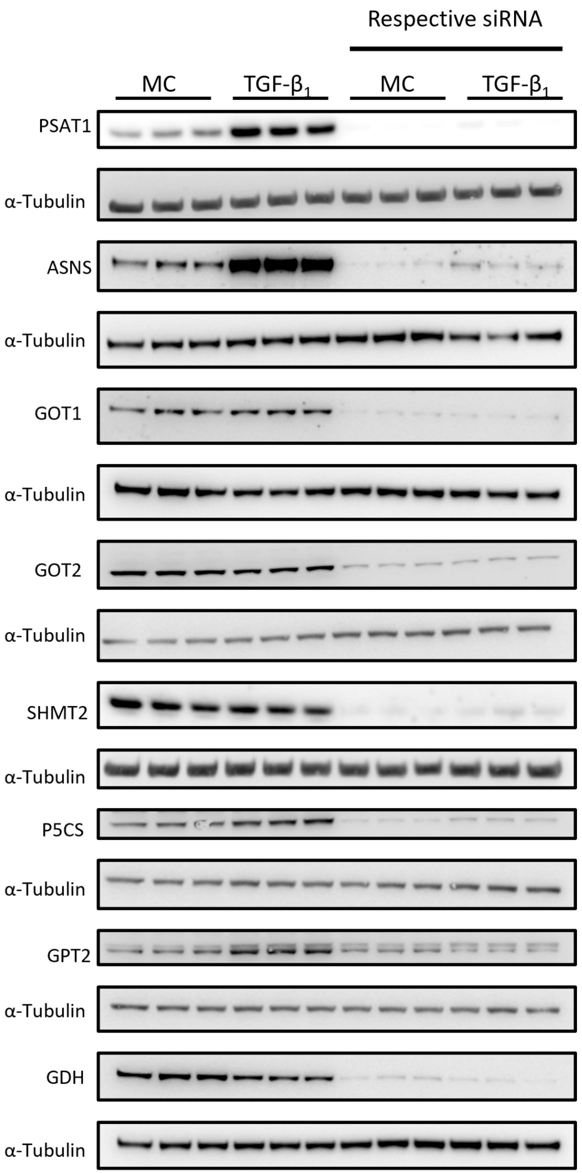

GPT2

HPA051514

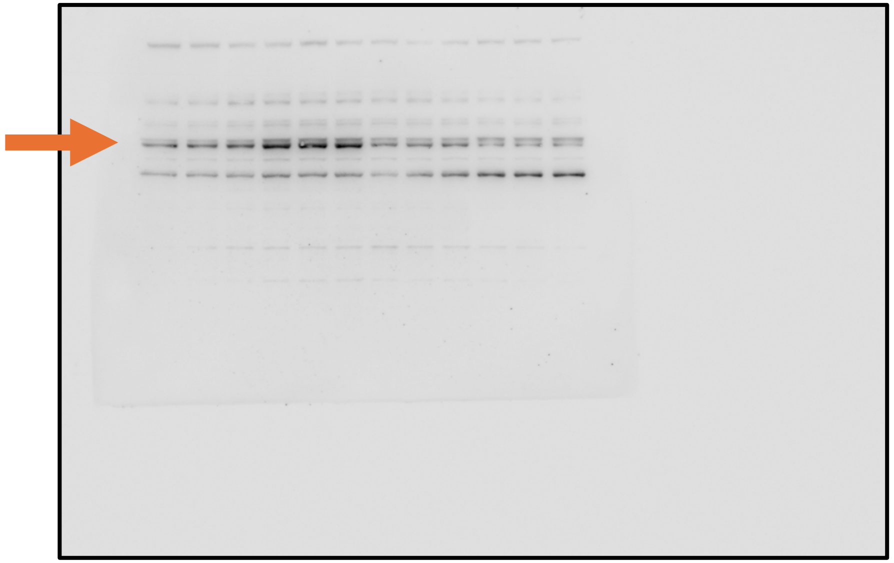

α-Tubulin

CST#9099

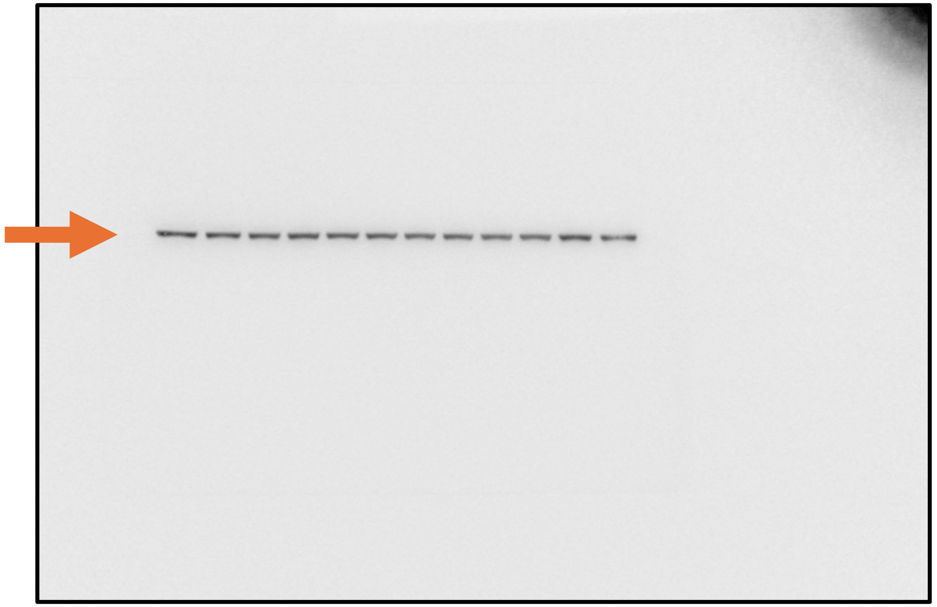

Full unedited gels for Fig s2b

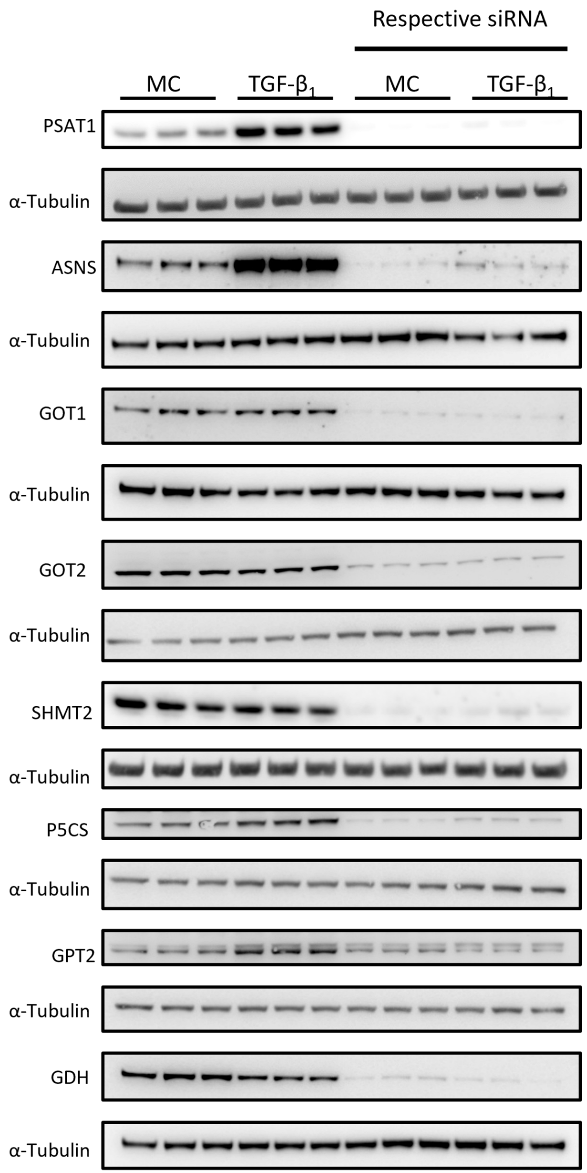

GDH

14299-1-AP

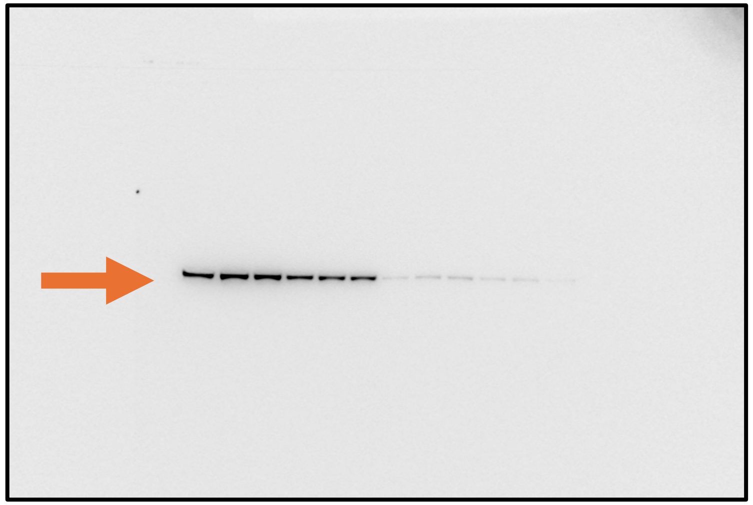

α-Tubulin

CST#9099

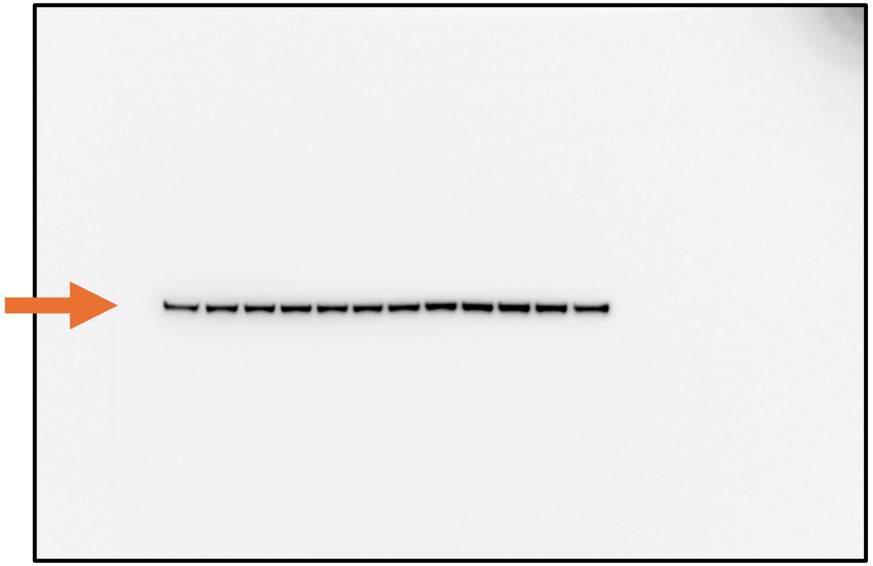

Full unedited gels for Fig s2b

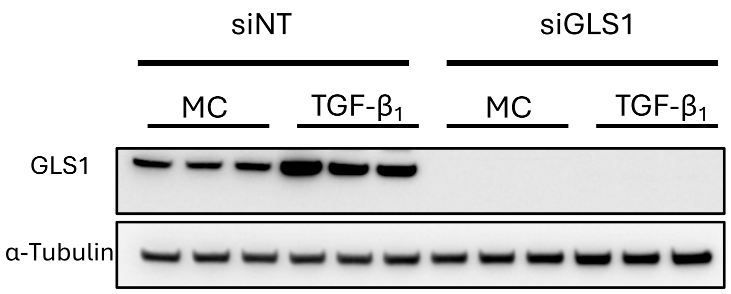

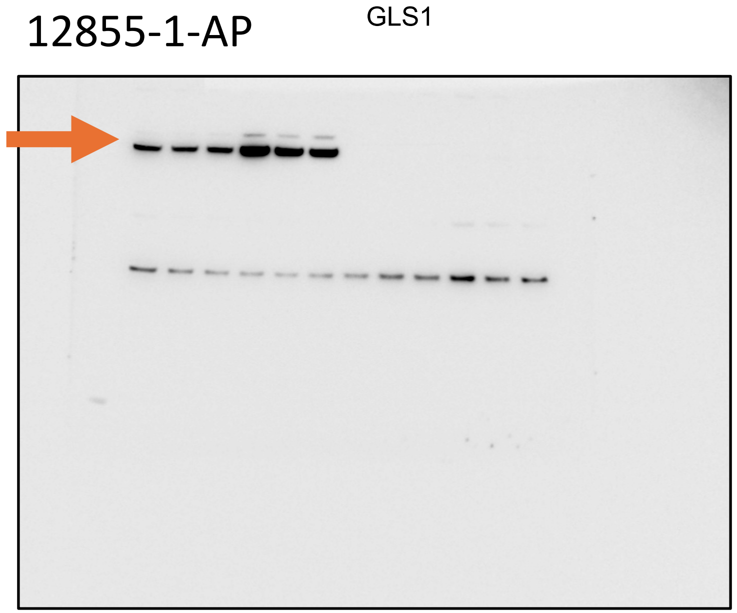

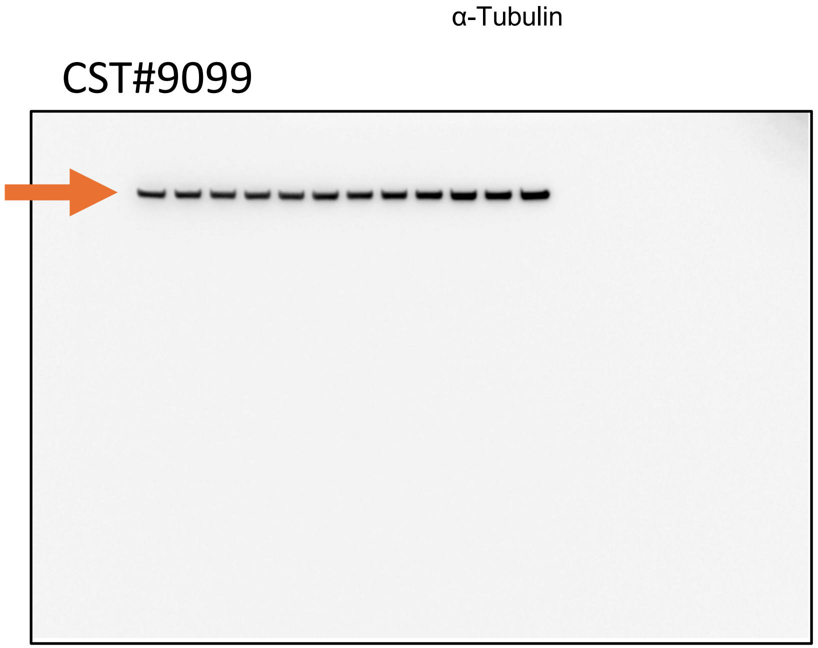

Full unedited gels for Fig s2h
